# Supplementary material for: Bioactive Polyketides from the Natural Complex of the Sea Urchin-Associated Fungi Penicillium sajarovii KMM 4718 and Aspergillus protuberus KMM 4747
Source: Int J Mol Sci. 2023 Nov 21;24(23):16568. doi: 10.3390/ijms242316568 (PMC10706121; doi:10.3390/ijms242316568)
Supplement: Supplementary file 1 [file ijms-24-16568-s001.zip › ijms-2719586-supplementary.pdf]

Supporting Information

Bioactive Polyketides from the Natural Complex of the Sea Urchin-associated Fungi *Penicillium sajarovii* KMM 4718 and *Aspergillus protuberus* KMM 4747

Elena V. Leshchenko [1,2,*], Dmitrii V. Berdyshev [1], Ekaterina A. Yurchenko [1,*], Alexandr S. Antonov [1], Gleb V. Borkunov [1,2], Natalya N. Kirichuk [1], Viktoria E. Chausova [1], Anatoly I. Kalinovskiy [1], Roman S. Popov [1], Yuliya V. Khudyakova [1], Ekaterina A. Chingizova [1], Artur R. Chingizov [1], Marina P. Isaeva [1] and Anton N. Yurchenko [1]

1 G.B. Elyakov Pacific Institute of Bioorganic Chemistry, Far Eastern Branch of the Russian Academy of Sciences, 159 Prospect 100-letiya Vladivostoka, Vladivostok 690022, Russian Federation; berdyshev@piboc.dvo.ru (D.V.B.); antonov_as@piboc.dvo.ru (A.S.A.); sheflera@bk.ru (N.N.K.); v.chausova@gmail.com (V.E.C.); kaaniv@piboc.dvo.ru (A.I.K.); popov_rs@piboc.dvo.ru (R.S.P.); 161070uuu@mail.ru (Y.V.K.); martyyas@mail.ru (E.A.C.); chingizov_ar@piboc.dvo.ru (A.R.C.); issaeva@piboc.dvo.ru (M.P.I.); yurchenkoan@piboc.dvo.ru (A.N.Y.).

2 Institute of High Technologies and Advanced Materials, Far Eastern Federal University, Vladivostok 690922, Russian Federation; gborkunov@gmail.com (G.V.B).

* Correspondence: leshchenko.ev@dvfu.ru (E.V.L.); eyurch@piboc.dvo.ru (E.A.Y.).

Figure S1. HRESIMS for 1
Figure S2. ^{1}H NMR spectrum of 1 measured at 500 MHz in acetone-d_6
Figure S3. ^{13}C NMR spectrum of 1 measured at 125 MHz in acetone-d_6
Figure S4. DEPT-135 spectrum of 1 measured at 125 MHz in acetone-d_6
Figure S5. HSQC spectrum of 1 measured in acetone-d_6
Figure S6. COSY spectrum of 1 measured in acetone-d_6
Figure S7. HMBC spectrum of 1 measured in acetone-d_6
Figure S8. HRESIMS for 2
Figure S9. ^{1}H NMR spectrum of 2 measured at 500 MHz in acetone-d_6
Figure S10. ^{13}C NMR spectrum of 2 measured at 125 MHz in acetone-d_6
Figure S11. DEPT-135 spectrum of 2 measured at 125 MHz in acetone-d_6
Figure S12. HSQC spectrum of 2 measured in acetone-d_6
Figure S13. COSY spectrum of 2 measured in acetone-d_6
Figure S14. HMBC spectrum of 2 measured in acetone-d_6
Figure S15. HRESIMS for 3
Figure S16. ^{1}H NMR spectrum of 3 measured at 500 MHz in acetone-d_6
Figure S17. ^{13}C NMR spectrum of 3 measured at 125 MHz in acetone-d_6
Figure S18. HRESIMS for 4
Figure S19. ^{1}H NMR spectrum of 4 measured at 500 MHz in acetone-d_6
Figure S20. ^{13}C NMR spectrum of 4 measured at 125 MHz in acetone-d_6
Figure S21. HRESIMS for 5

Figure S22. ^{1}H NMR spectrum of 5 measured at 500 MHz in acetone-d_6
Figure S23. ^{13}C NMR spectrum of 5 measured at 125 MHz in acetone-d_6
Figure S24. HRESIMS for 6
Figure S25. ^{1}H NMR spectrum of 6 measured at 300 MHz in acetone-d_6
Figure S26. ^{13}C NMR spectrum of 6 measured at 75 MHz in acetone-d_6
Figure S27. HRESIMS for 7
Figure S28. ^{1}H NMR spectrum of 7 measured at 700 MHz in acetone-d_6
Figure S29. ^{13}C NMR spectrum of 7 measured at 175 MHz in acetone-d_6
Figure S30. HRESIMS for 8
Figure S31. ^{1}H NMR spectrum of 8 measured at 700 MHz in acetone-d_6
Figure S32. ^{13}C NMR spectrum of 8 measured at 175 MHz in acetone-d_6
Figure S33. HRESIMS for 9
Figure S34. ^{1}H NMR spectrum of 9 measured at 700 MHz in $CDCl_3$
Figure S35. ^{13}C NMR spectrum of 9 measured at 175 MHz in $CDCl_3$
Figure S36. HRESIMS for 10
Figure S37. ^{1}H NMR spectrum of 10 measured at 500 MHz in acetone-d_6
Figure S38. ^{13}C NMR spectrum of 10 measured at 125 MHz in acetone-d_6
Figure S39. HRESIMS for 11
Figure S40. ^{1}H NMR spectrum of 11 measured at 500 MHz in acetone-d_6
Figure S41. ^{13}C NMR spectrum of 11 measured at 125 MHz in acetone-d_6
Figure S42. Two most stale conformations of *R*-1 calculated with B3LYP/cc-pvTz_PCM method.
Figure S43. The optimized structures of 4*R*-1&(CH_3OH)×2.

Figure S1. HRESIMS for 1

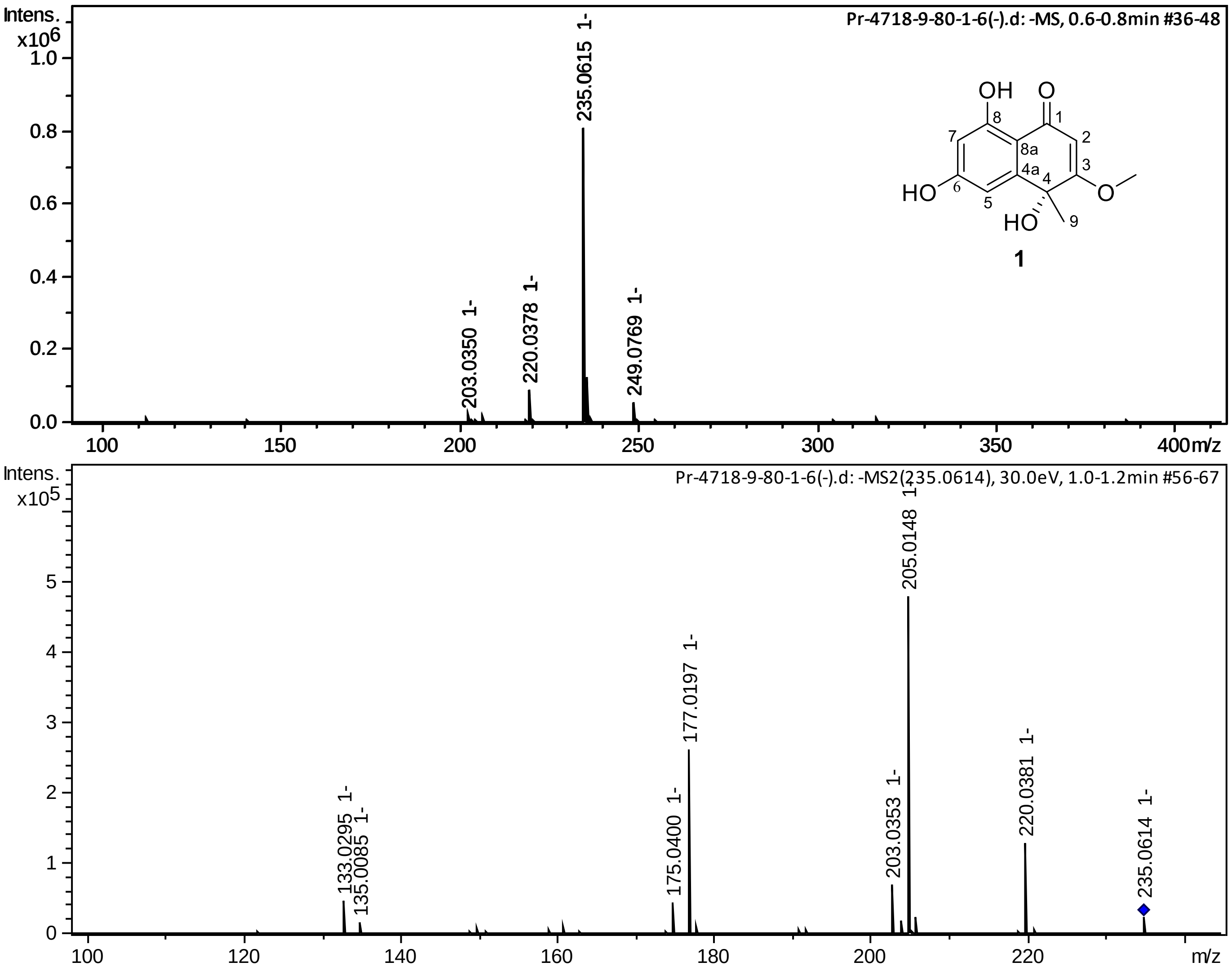

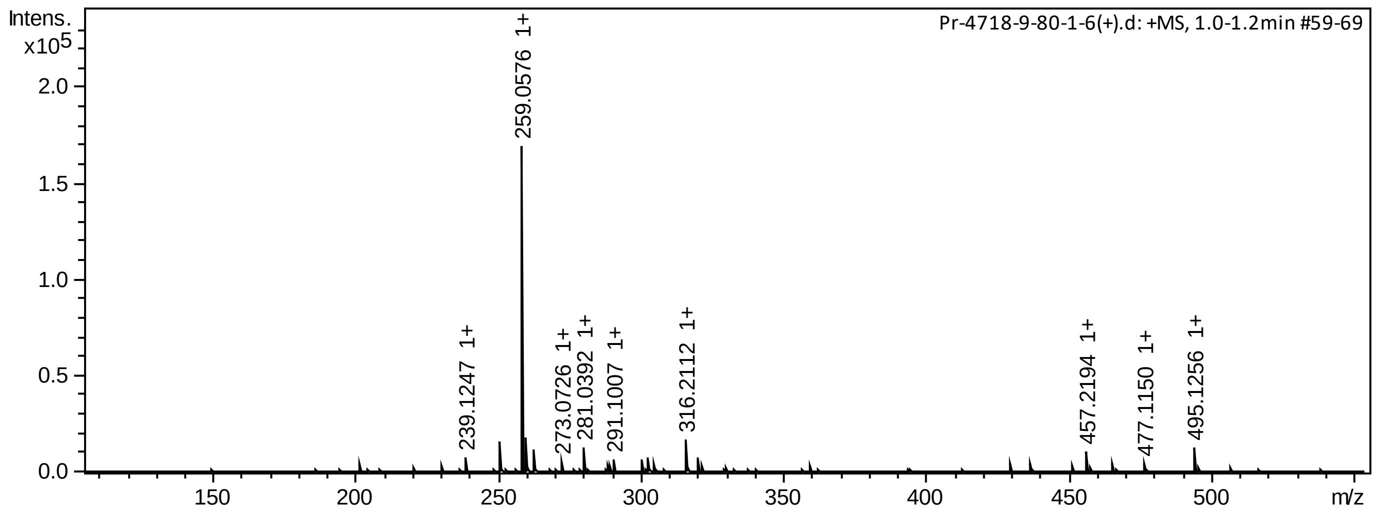
Intens.
x10^5
Pr-4718-9-80-1-6(+).d: +MS, 1.0-1.2min #59-69
259.0576 1+
239.1247 1+
273.0726 1+
281.0392 1+
291.1007 1+
316.2112 1+
457.2194 1+
477.1150 1+
495.1256 1+
m/z

Figure S2. ^{1}H NMR spectrum of 1 measured at 500 MHz in acetone-d_6

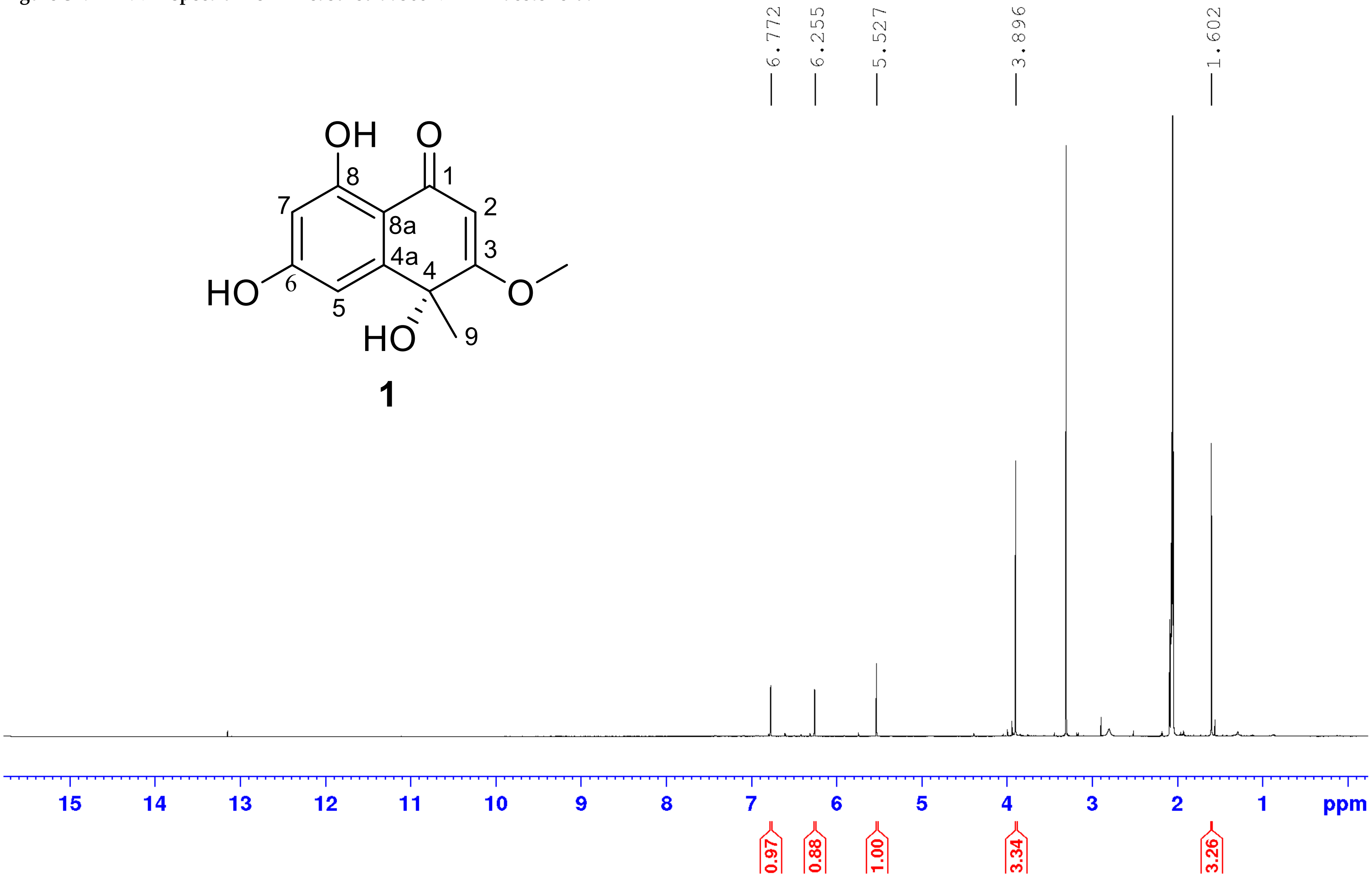

Figure S3. ^{13}C **NMR spectrum of 1 measured at 125 MHz in acetone-d**$_6$

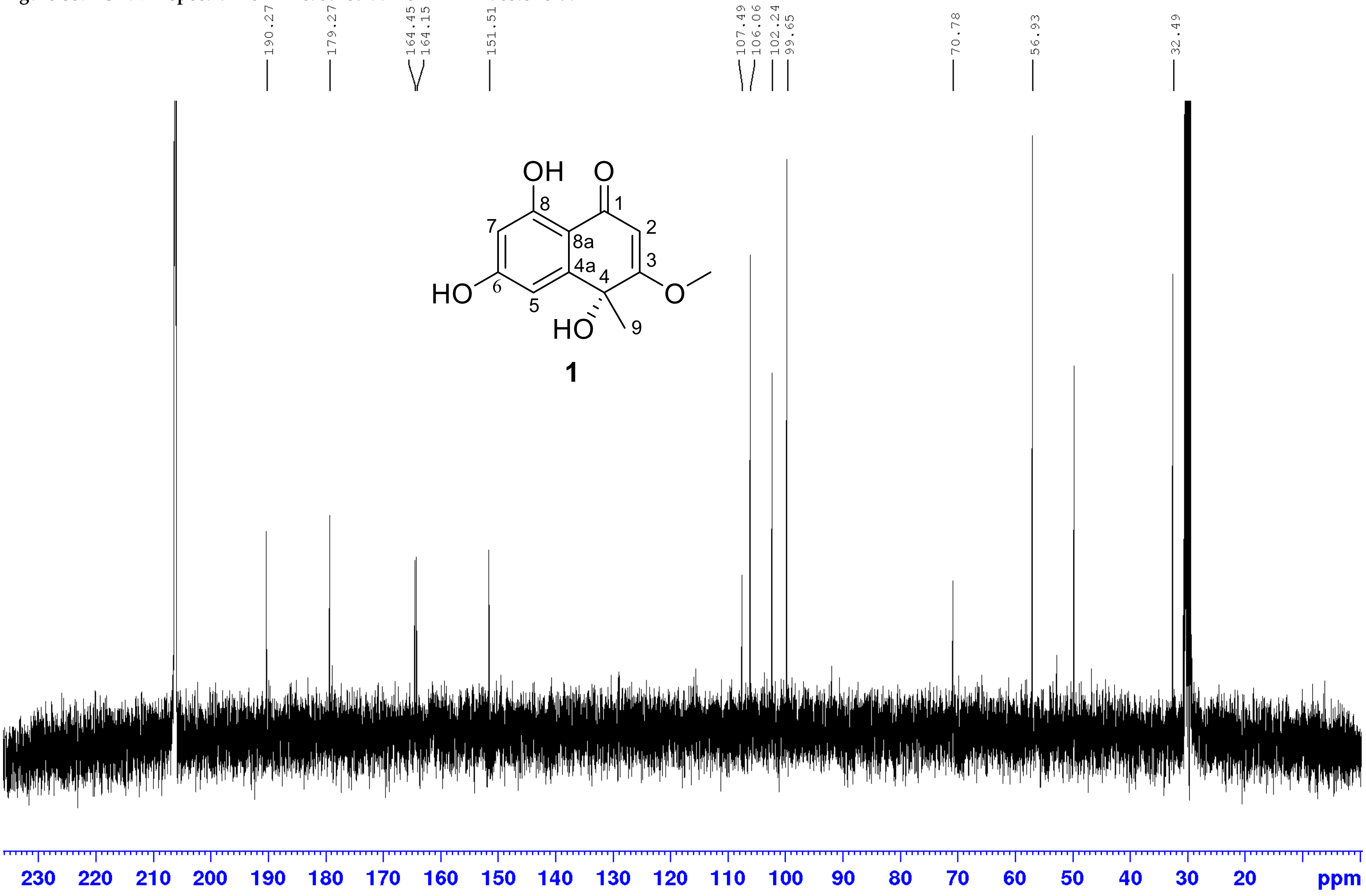

Figure S4. DEPT-135 spectrum of 1 measured at 125 MHz in acetone-d_6

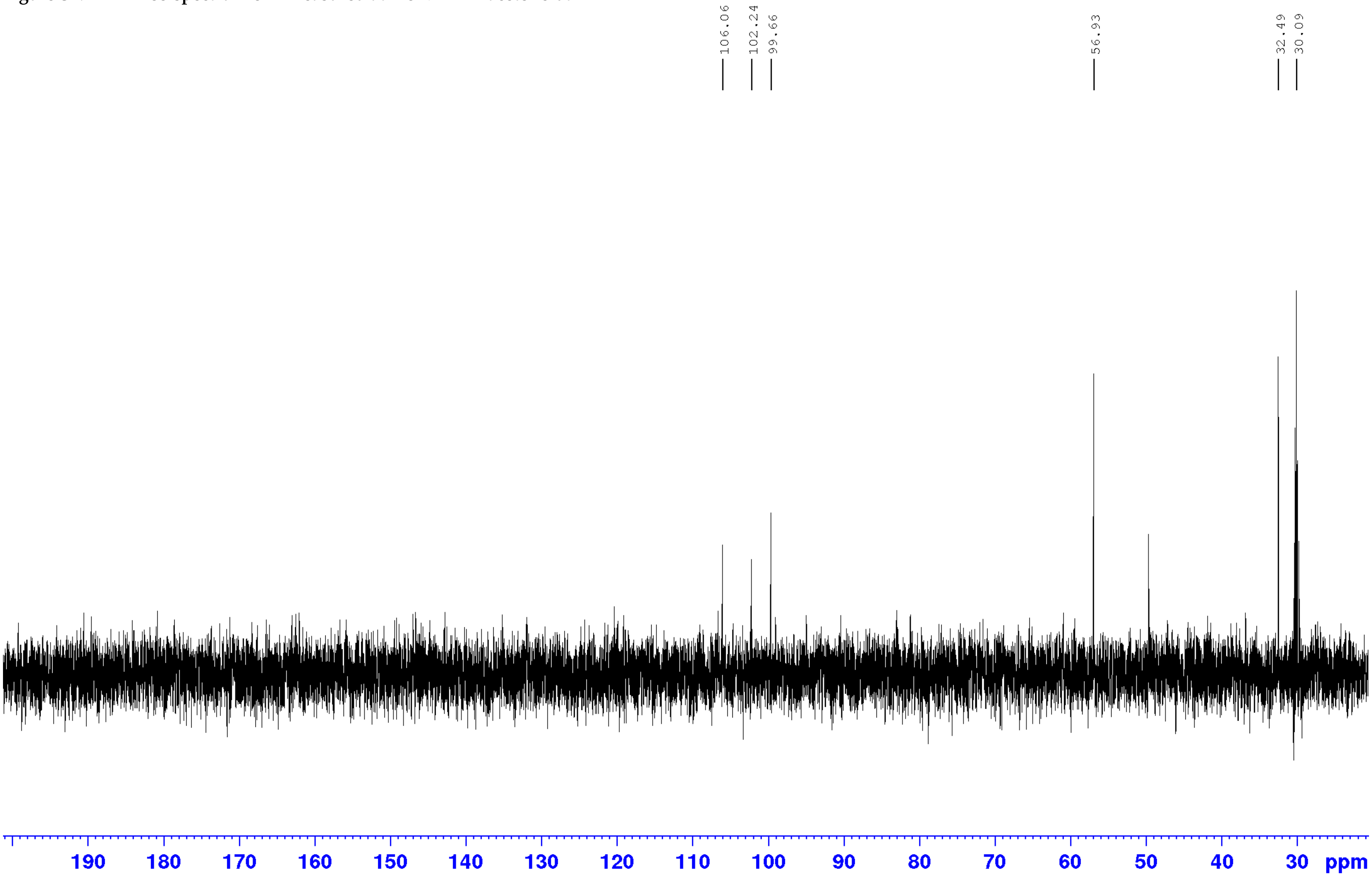

Figure S5. **HSQC spectrum of 1 measured in acetone-d$_6$**

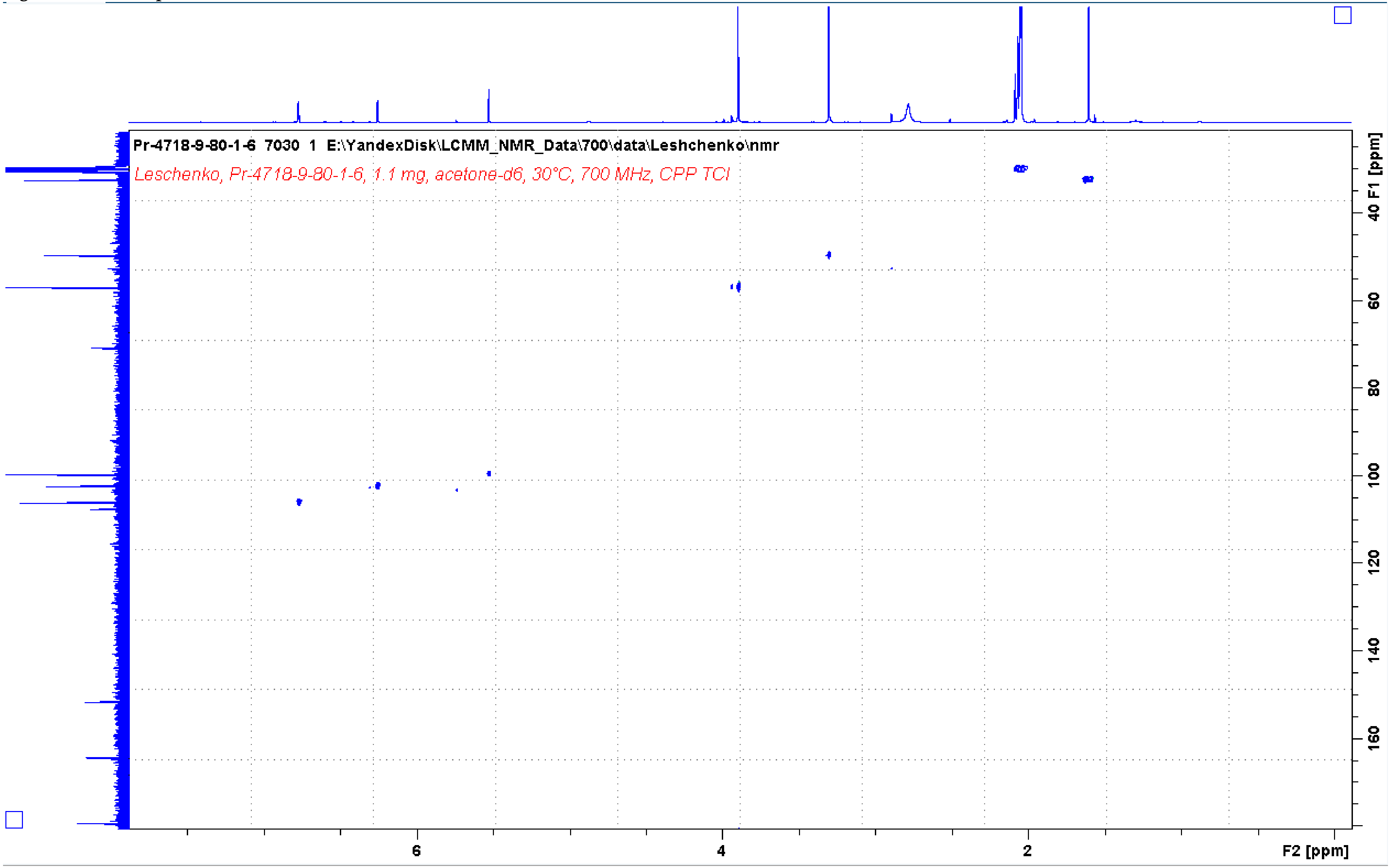

Figure S6. COSY spectrum of 1 measured in acetone-d_6

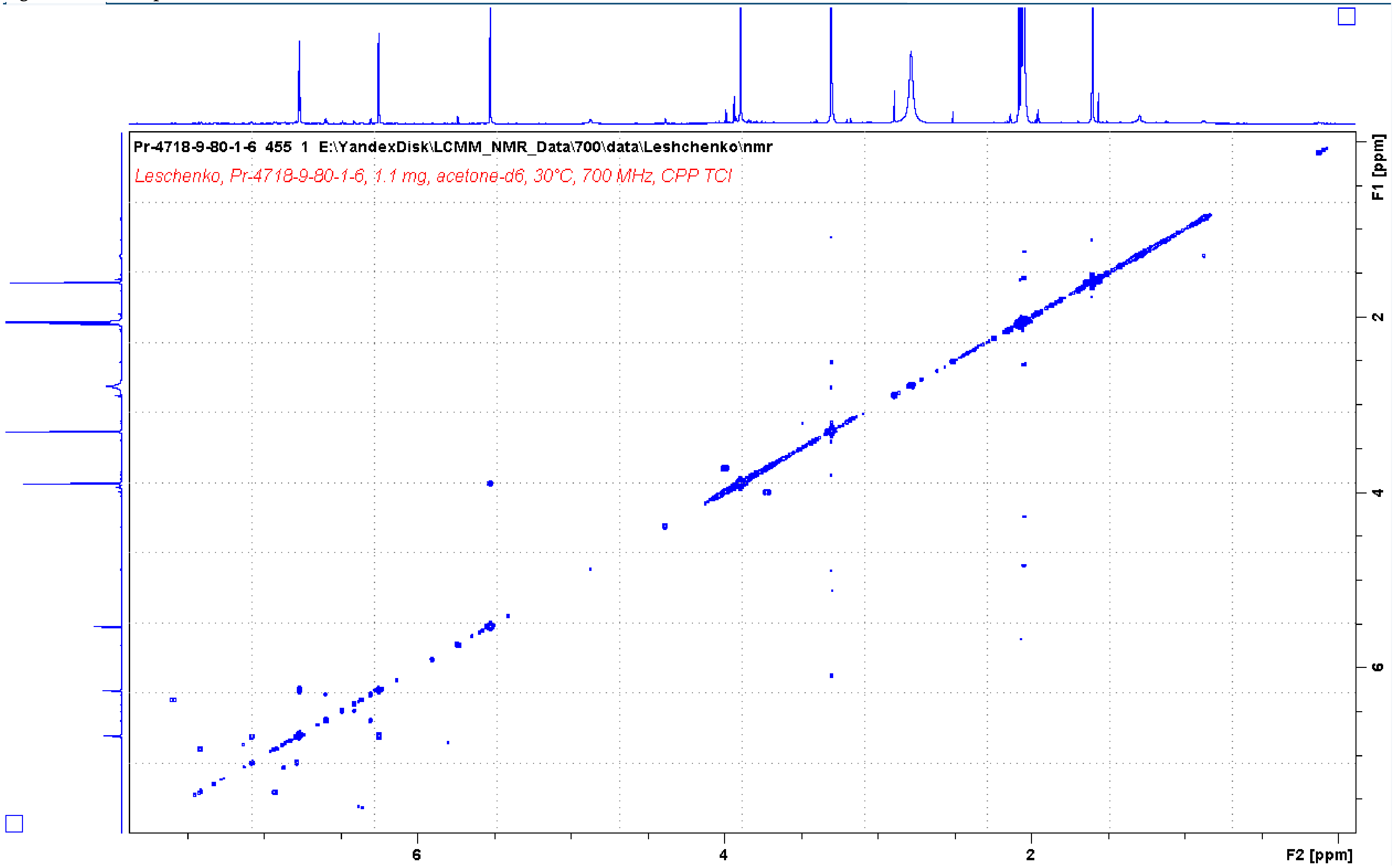

Figure S7. HMBC spectrum of 1 measured in acetone-d_6

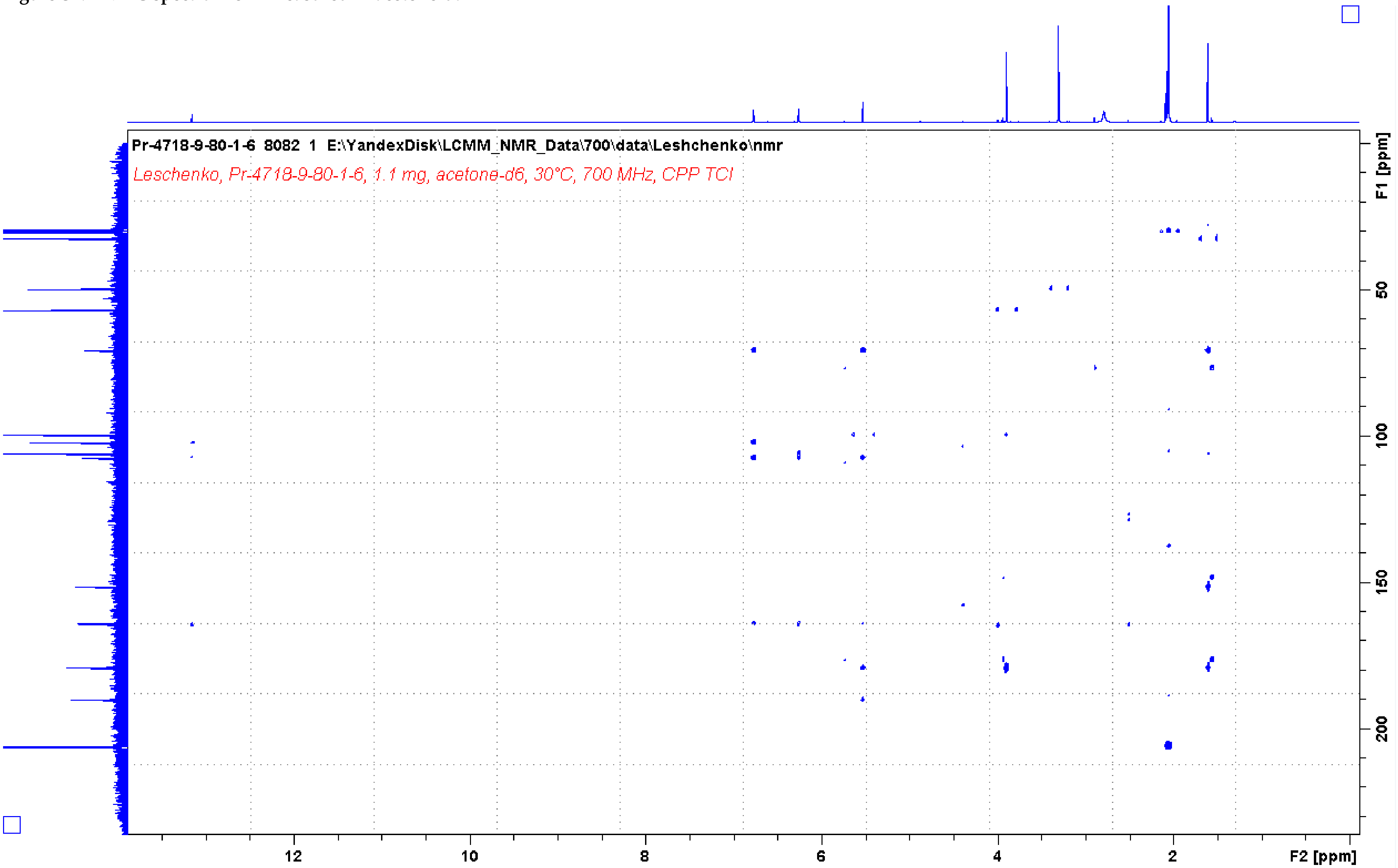

Figure S8. HRESIMS for 2

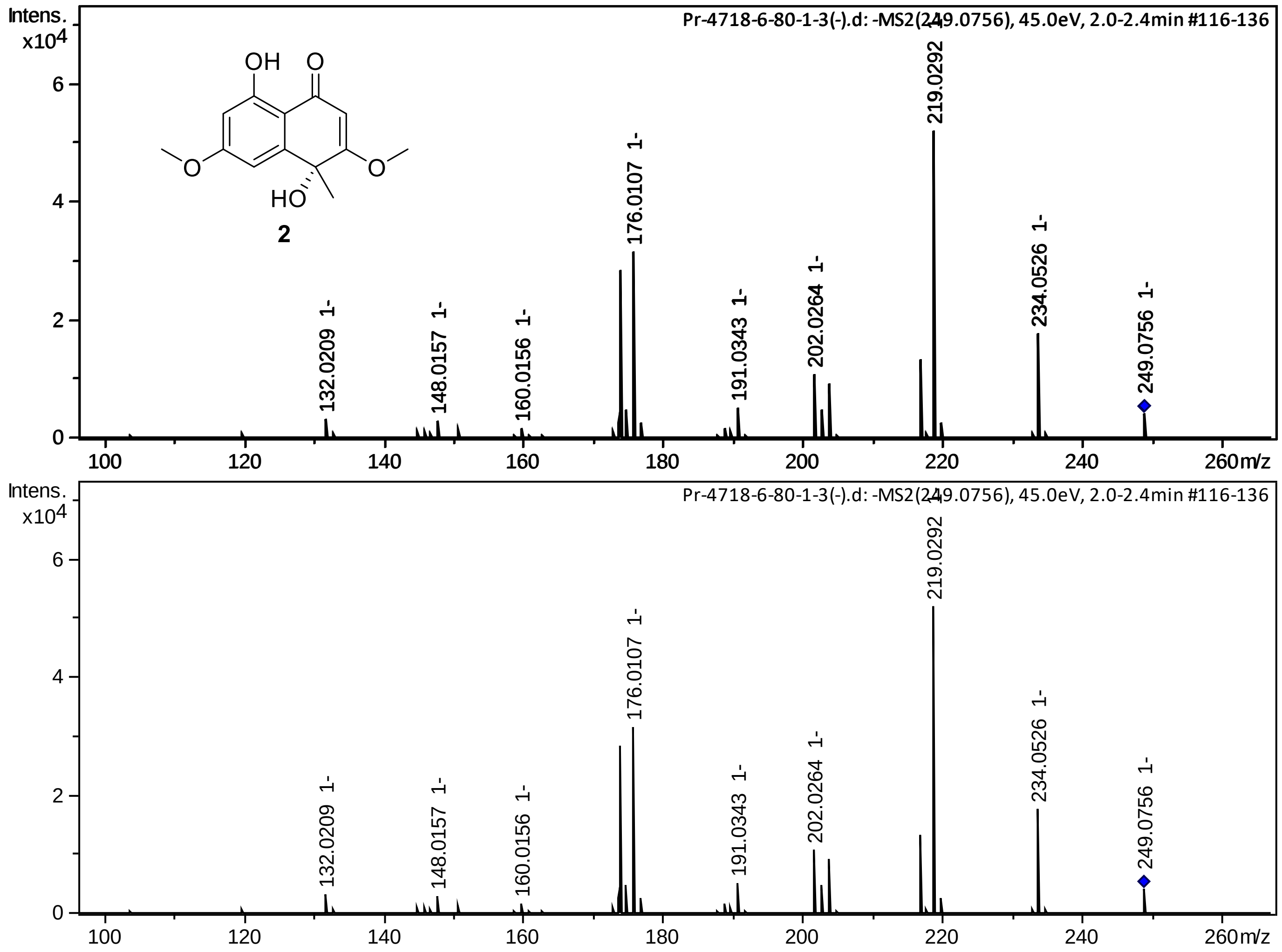

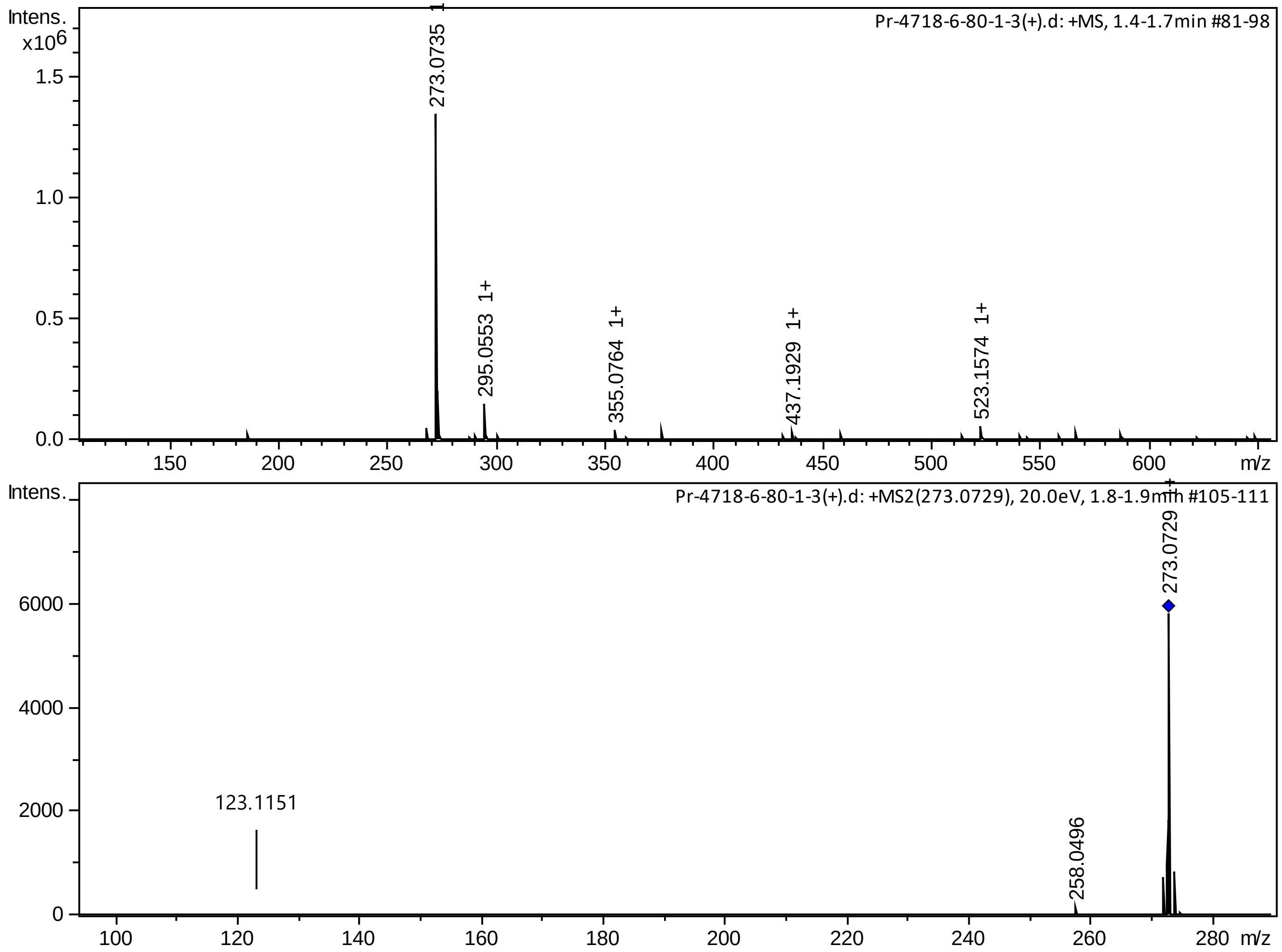
Intens.
x10^6
Pr-4718-6-80-1-3(+).d: +MS, 1.4-1.7min #81-98
273.0735
295.0553 1+
355.0764 1+
437.1929 1+
523.1574 1+
m/z
Intens.
Pr-4718-6-80-1-3(+).d: +MS2(273.0729), 20.0eV, 1.8-1.9min #105-111
273.0729
123.1151
258.0496
m/z

Figure S9. ^{1}H **NMR spectrum of 2 measured at 500 MHz in acetone-d**$_6$

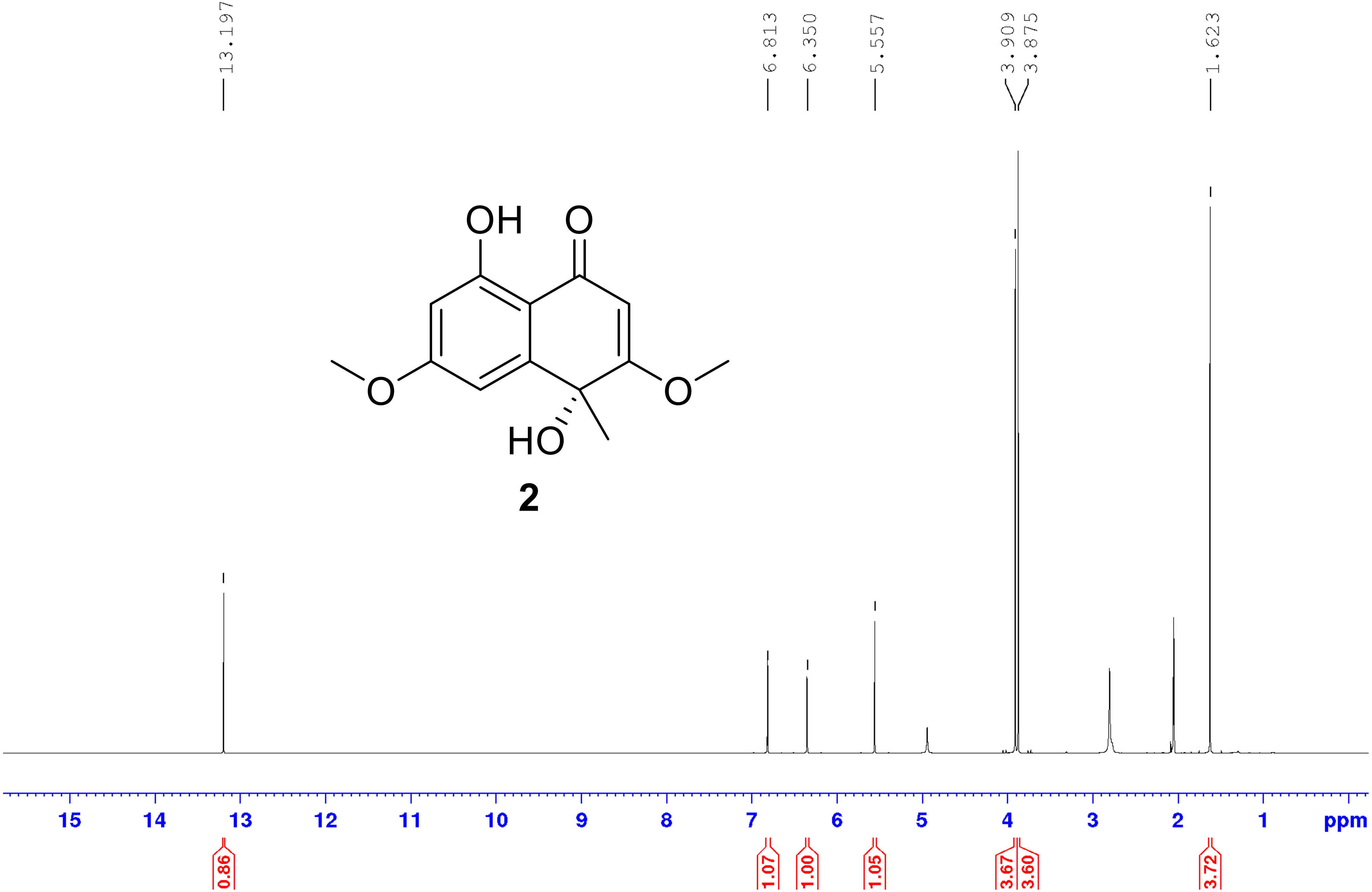

Figure S10. ^{13}C NMR spectrum of **2** measured at 125 MHz in acetone-d_6

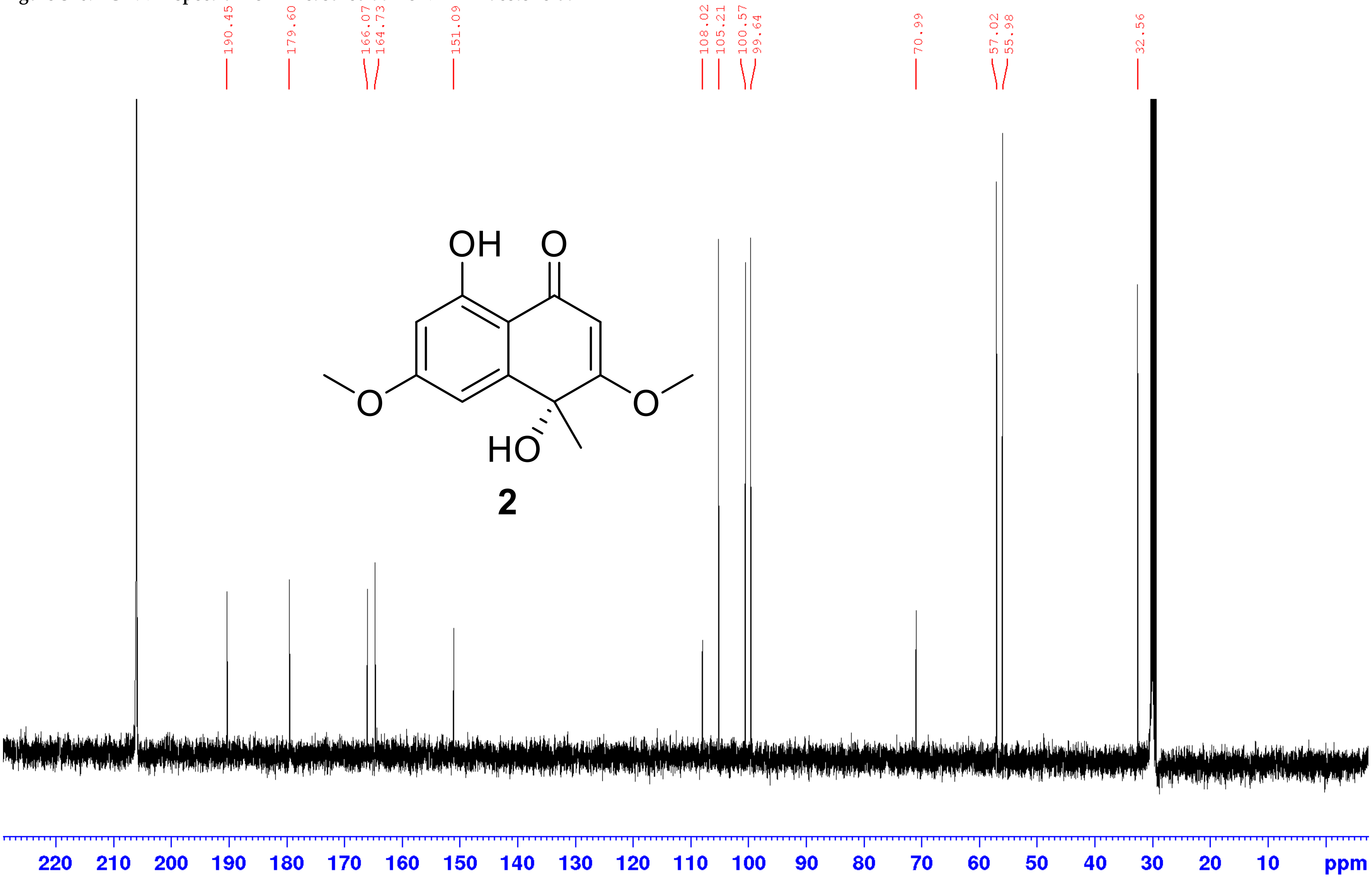

Figure S11. DEPT-135 spectrum of 2 measured at 125 MHz in acetone-d₆

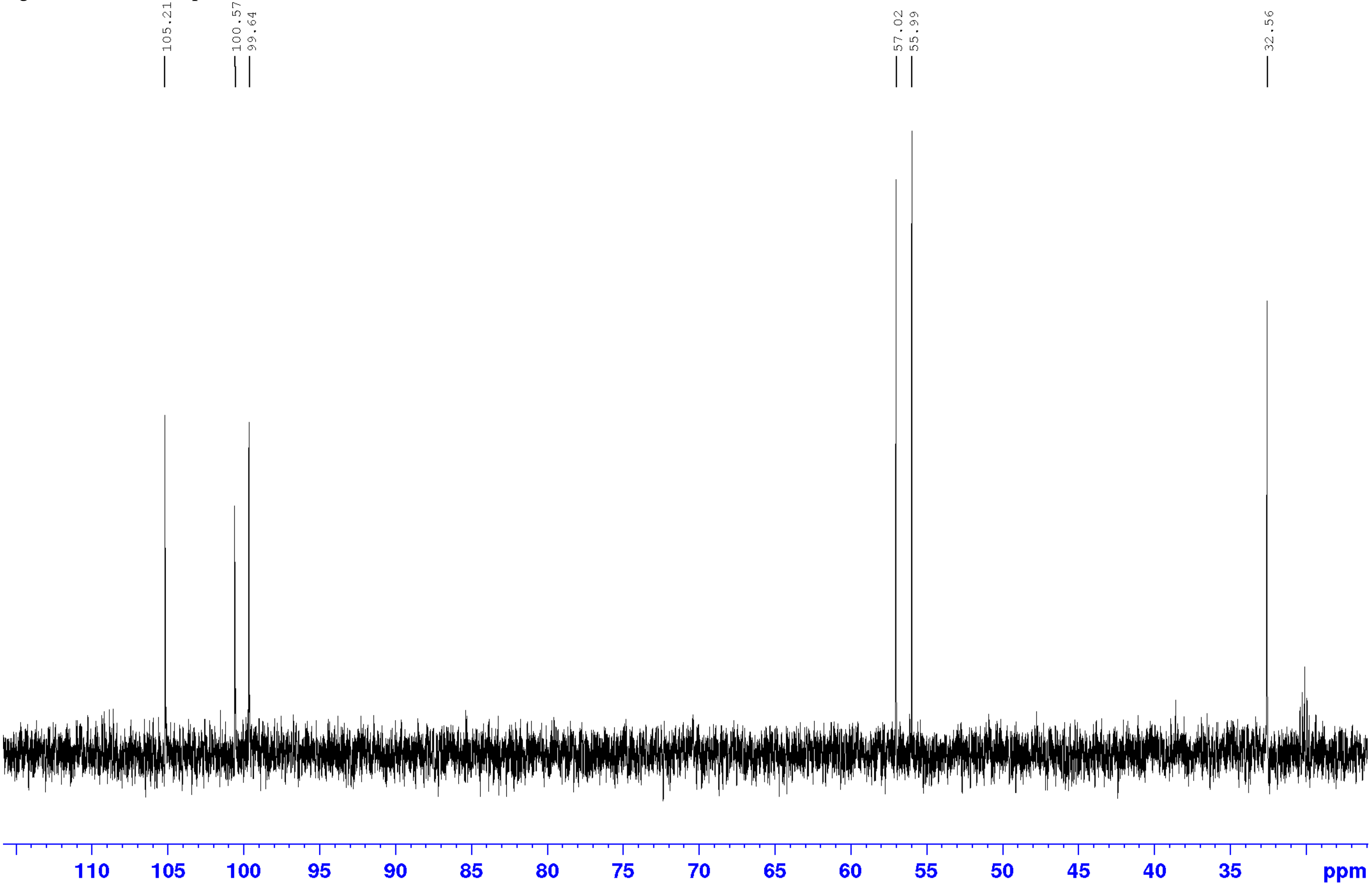

Figure S12. HSQC spectrum of 2 measured in acetone-d₆

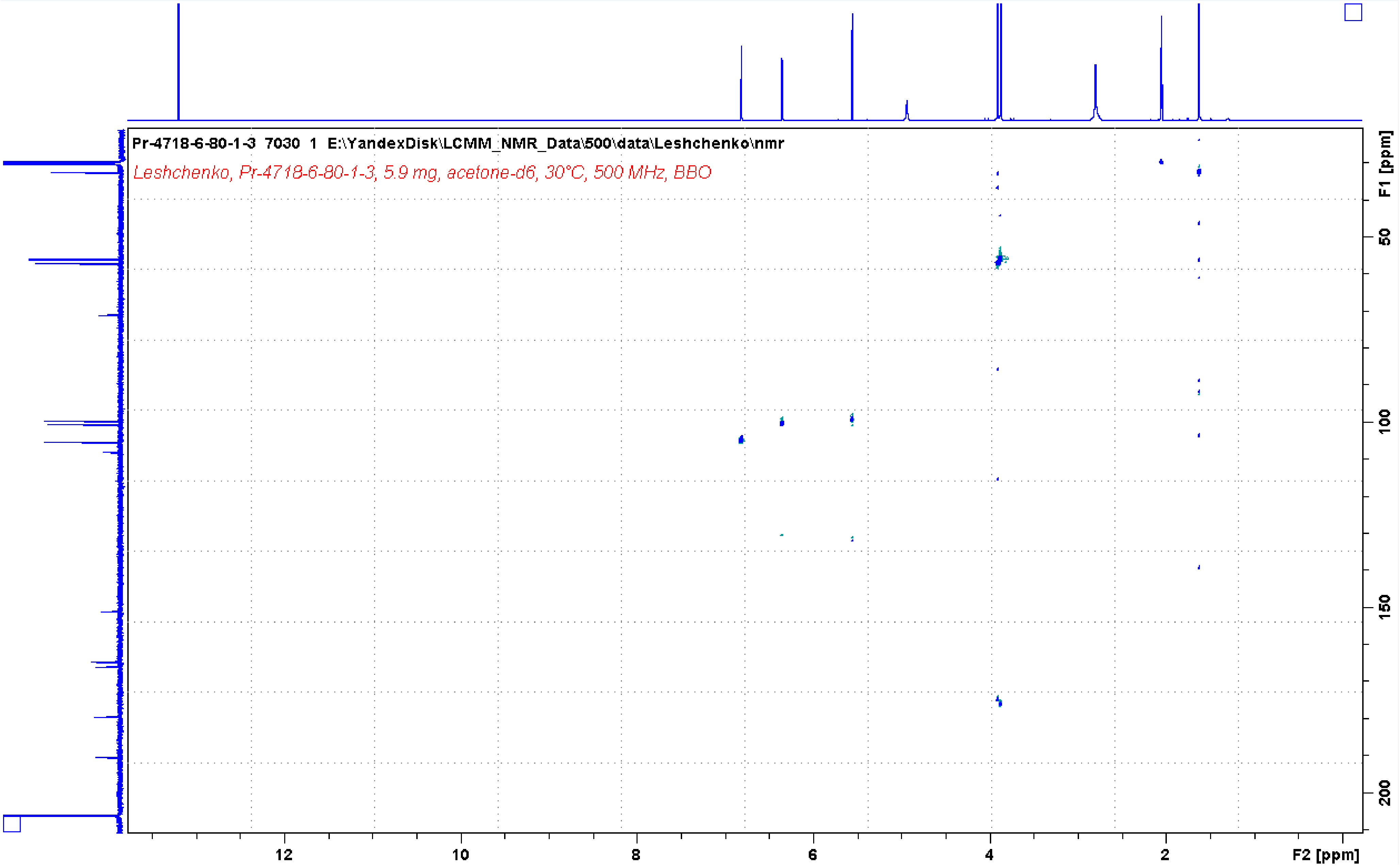

Figure S13. COSY spectrum of 2 measured in acetone-d_6

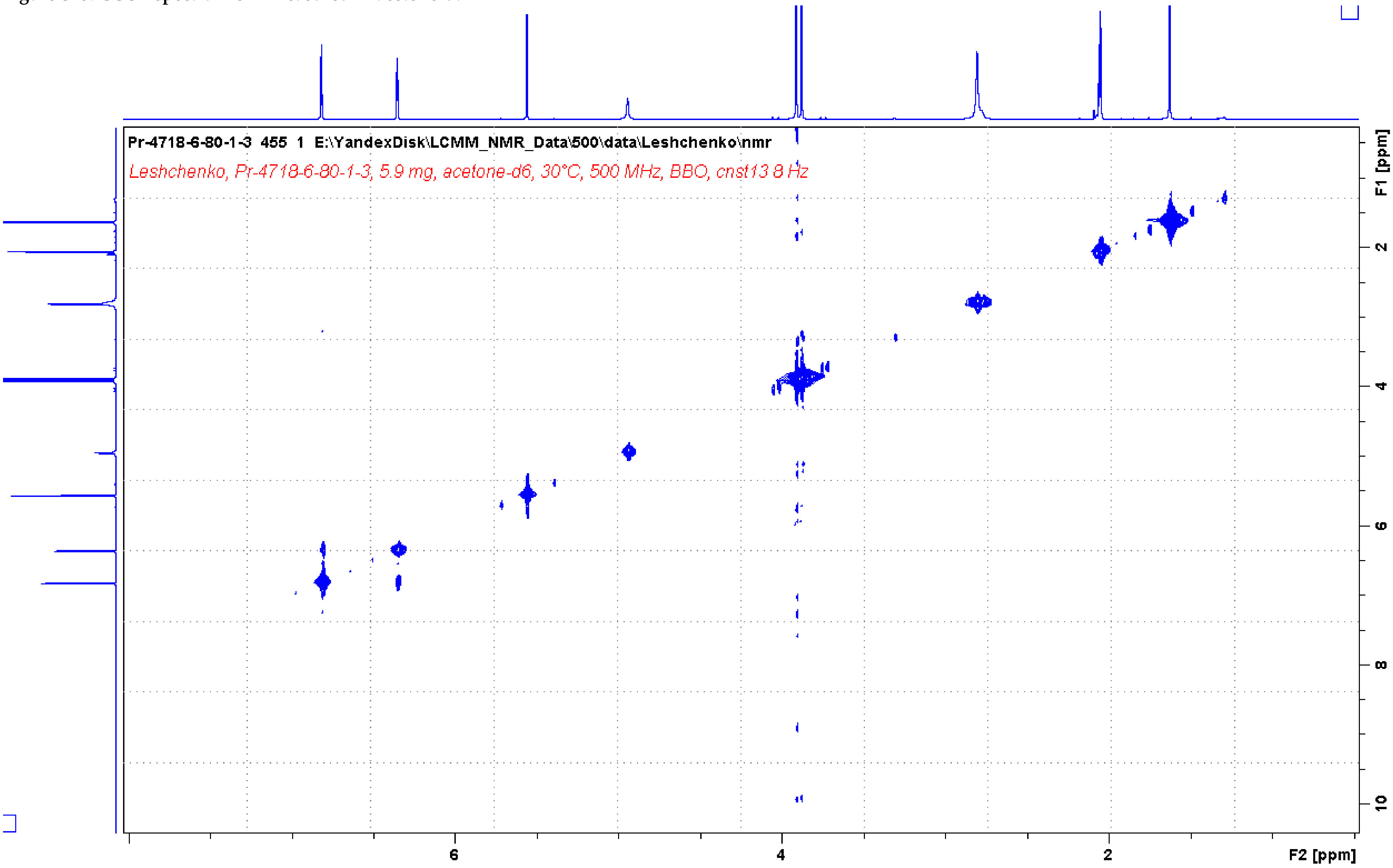

Figure S14. HMBC spectrum of 2 measured in acetone-d6

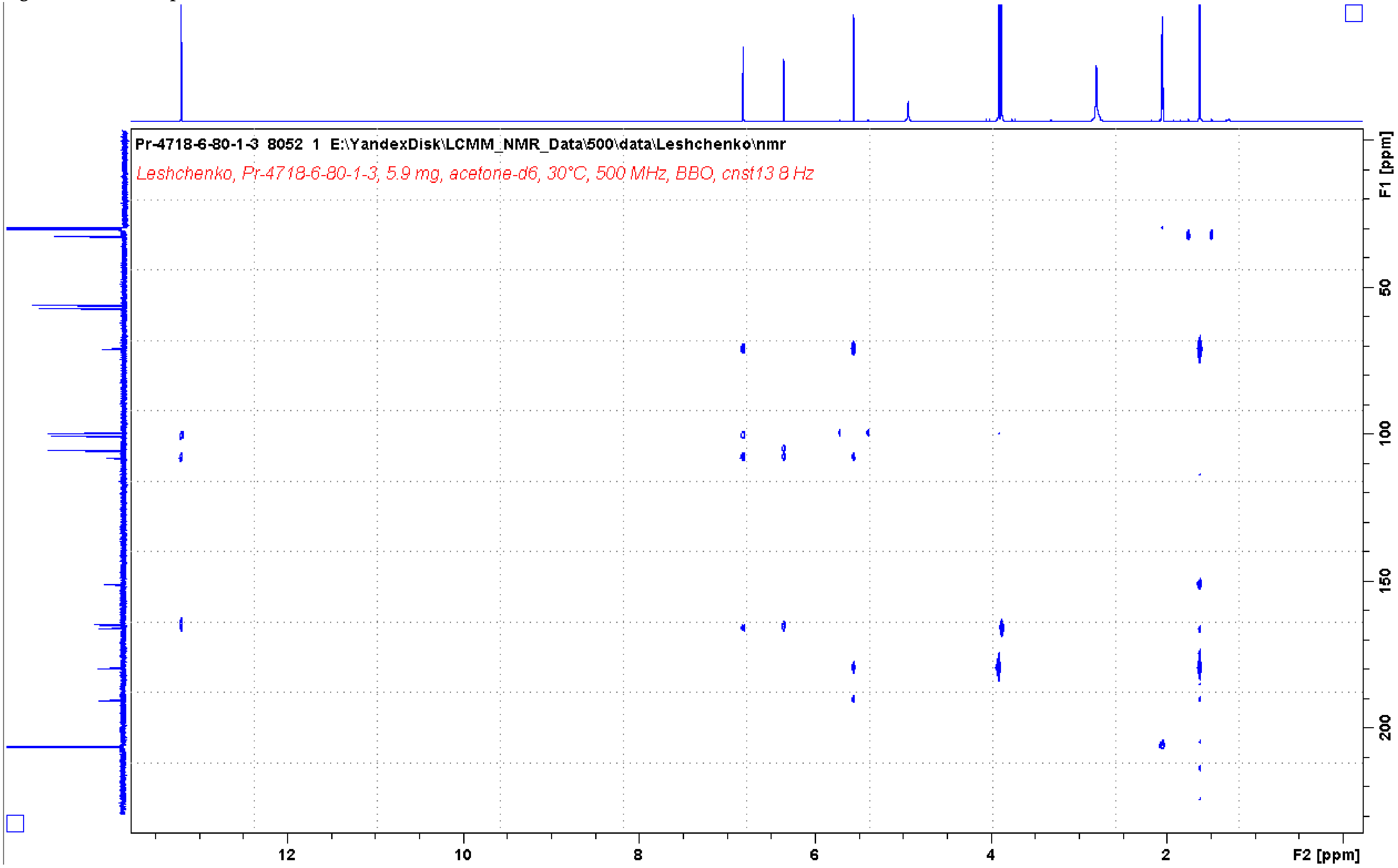

Figure S15. HRESIMS for 3

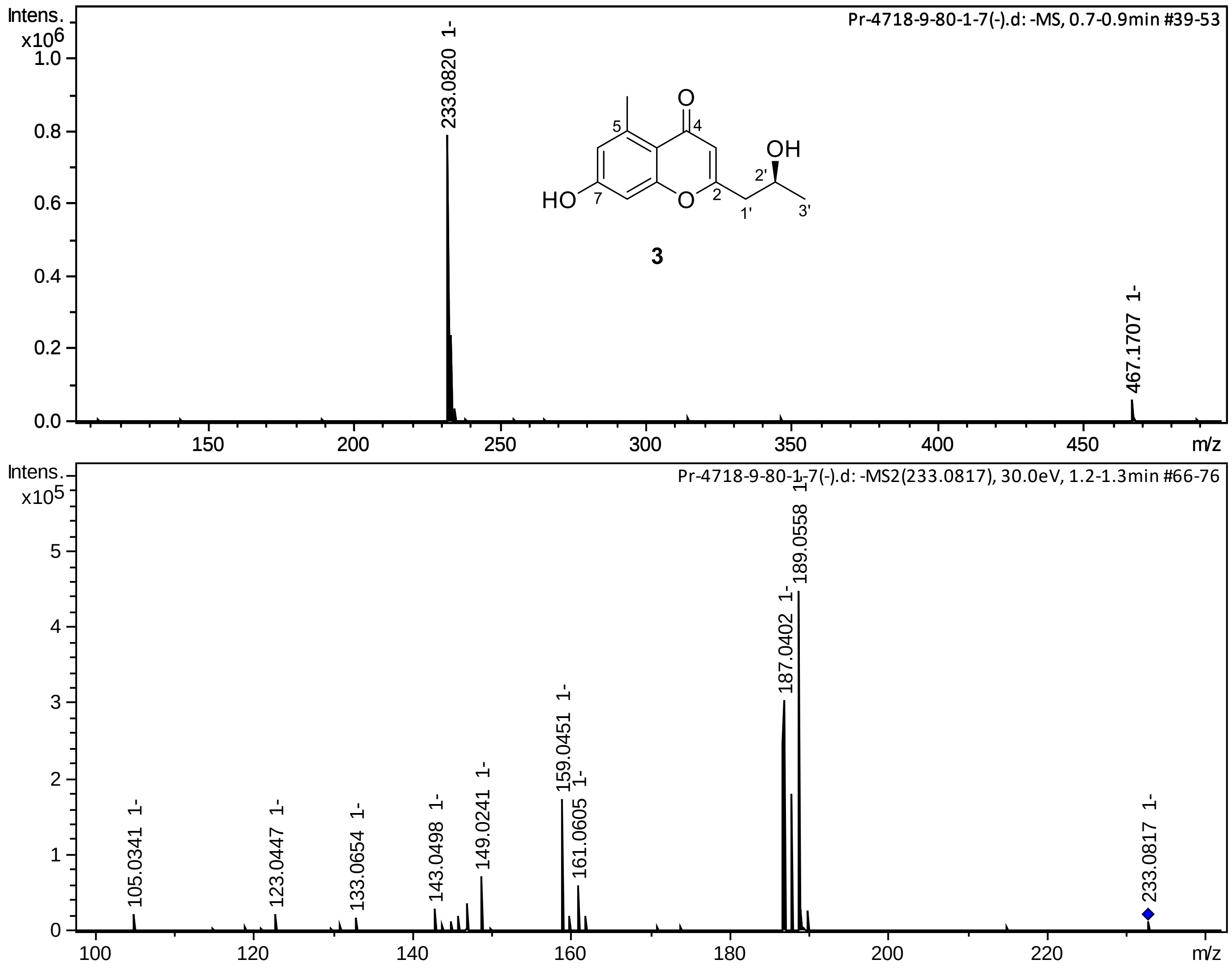

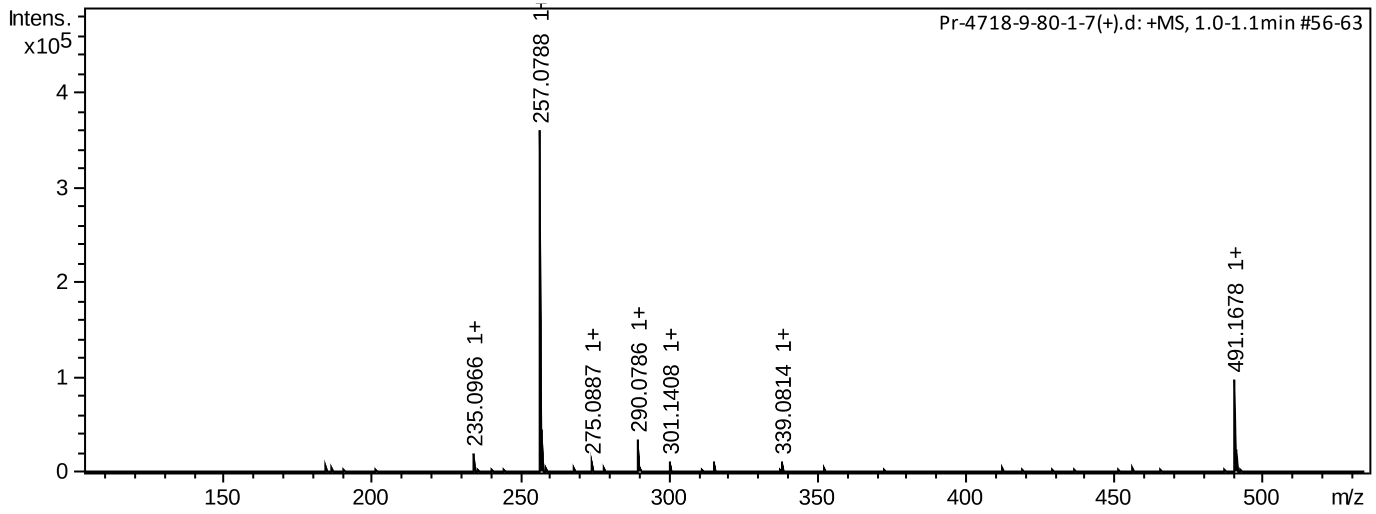
Intens.
x10^5
4
3
2
1
0
Pr-4718-9-80-1-7(+).d: +MS, 1.0-1.1min #56-63
257.0788 1+
235.0966 1+
275.0887 1+
290.0786 1+
301.1408 1+
339.0814 1+
491.1678 1+
150
200
250
300
350
400
450
500
m/z

Figure S16. ^{1}H NMR spectrum of 3 measured at 500 MHz in acetone-d_6

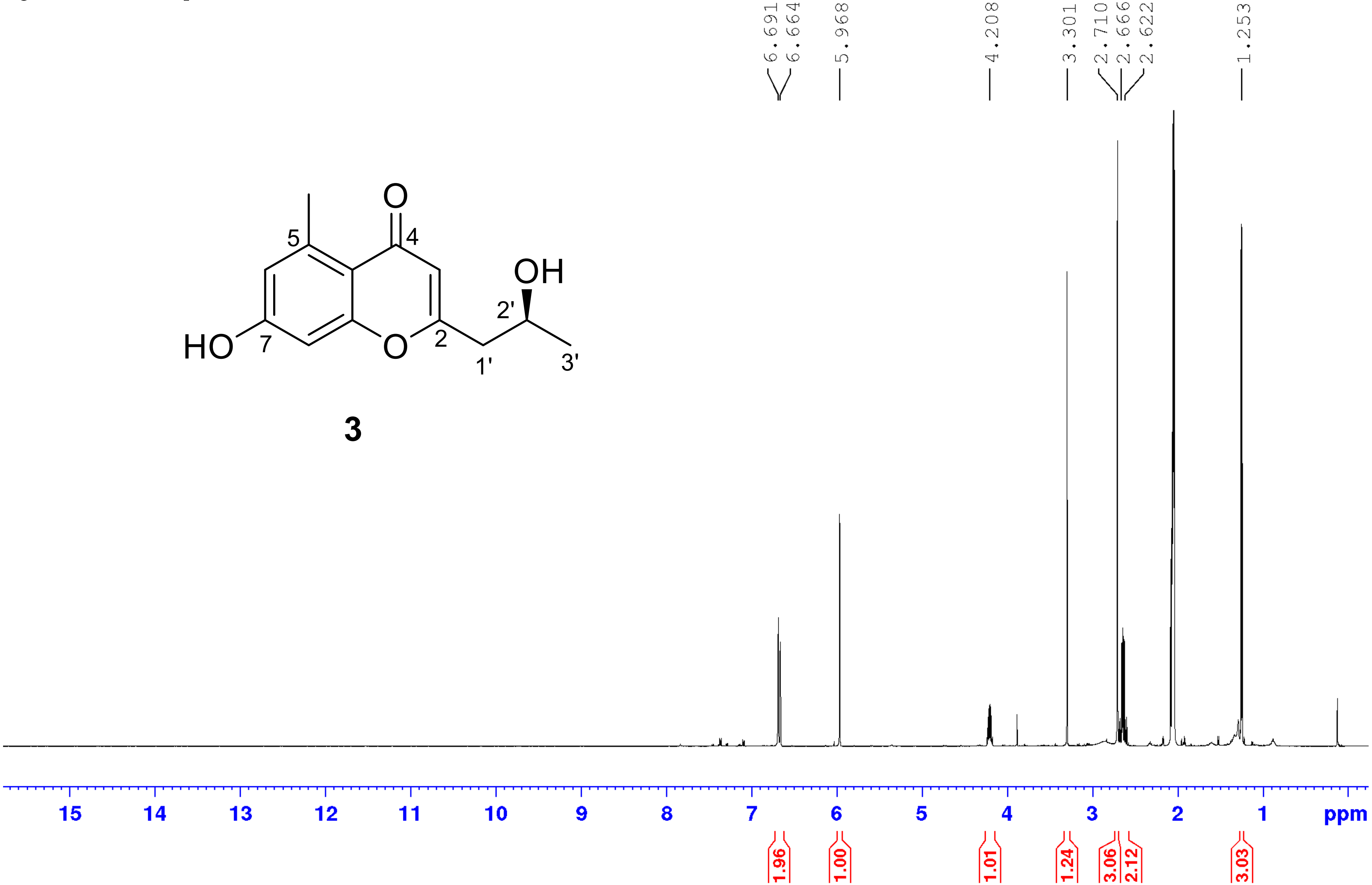

Figure S17. ^{13}C **NMR spectrum of 3 measured at 125 MHz in acetone-d_6**

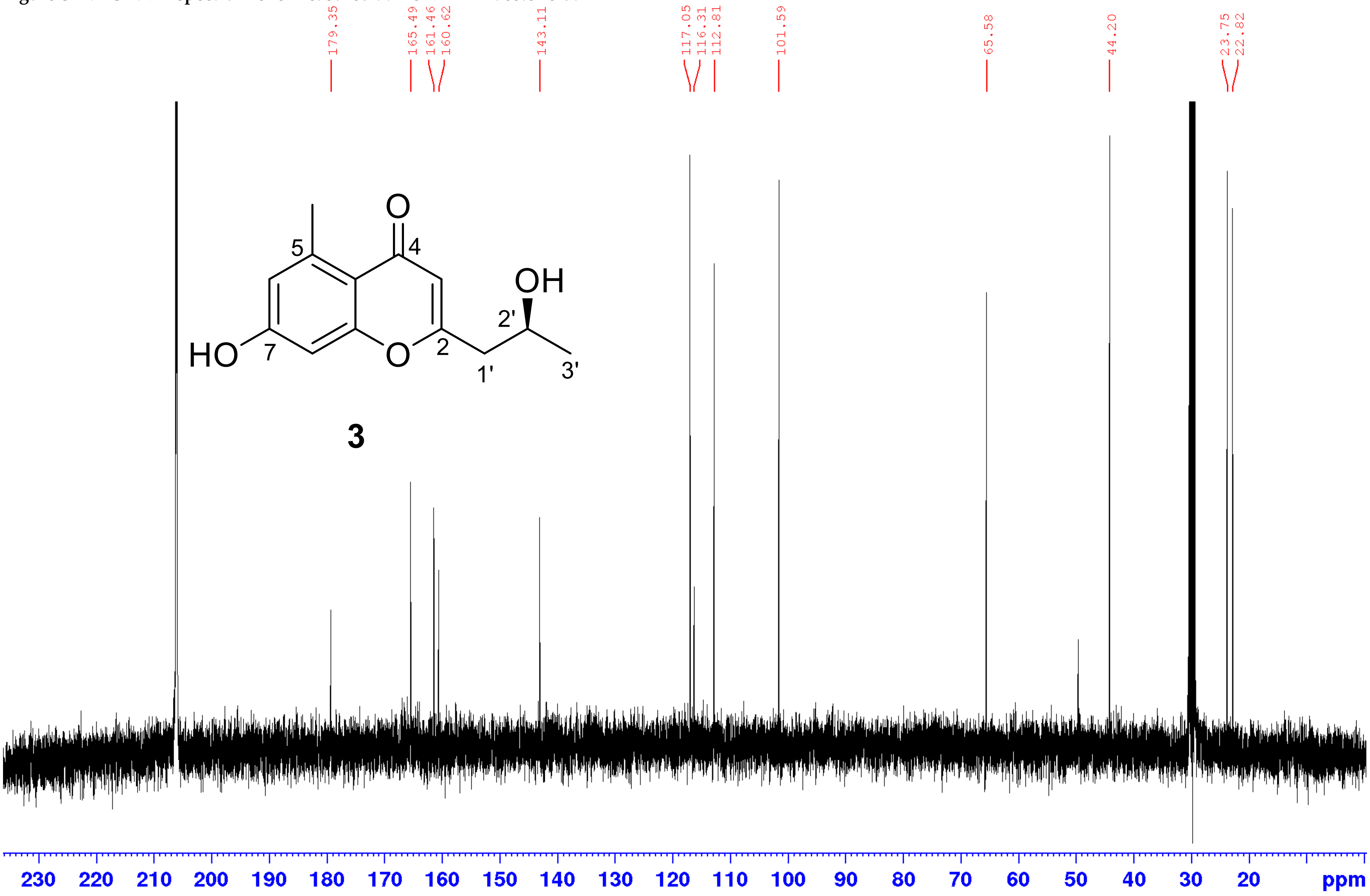

Figure S18. HRESIMS for 4

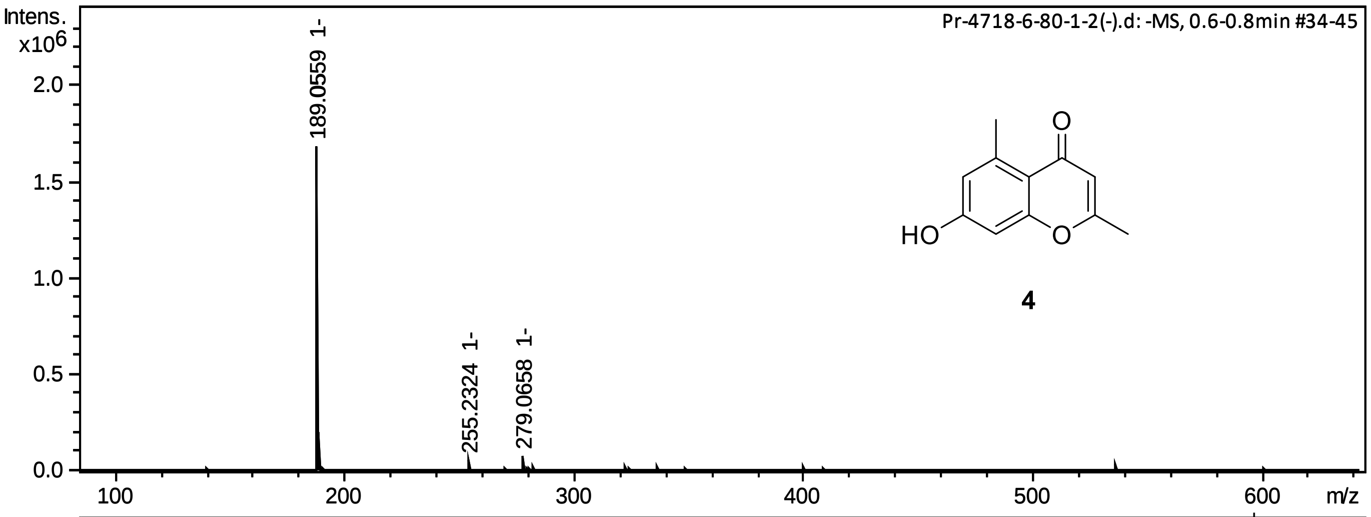

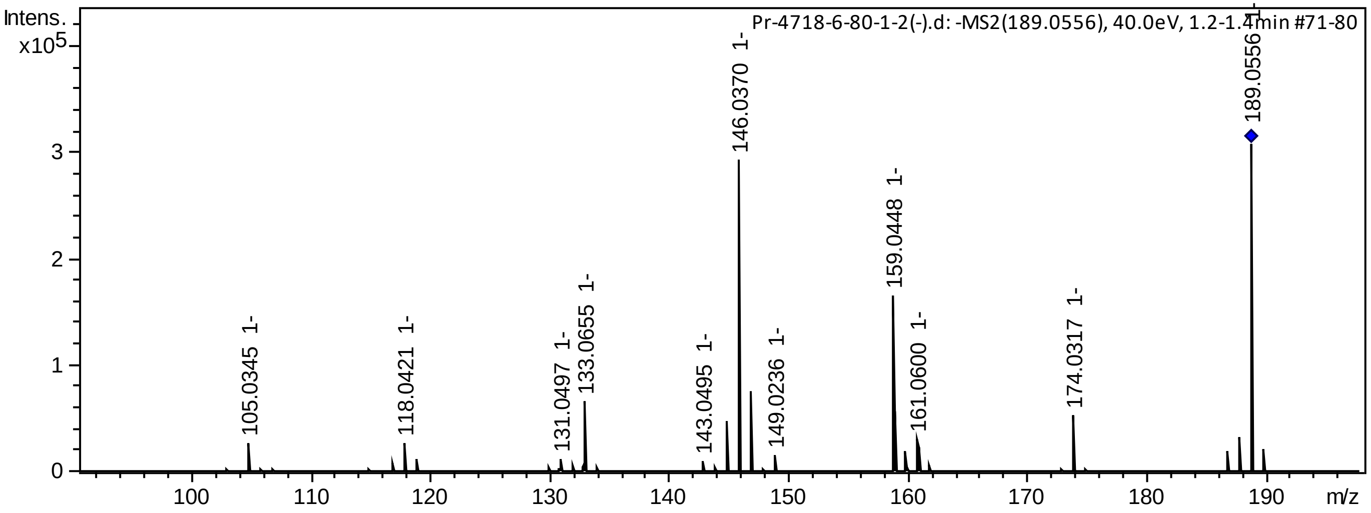

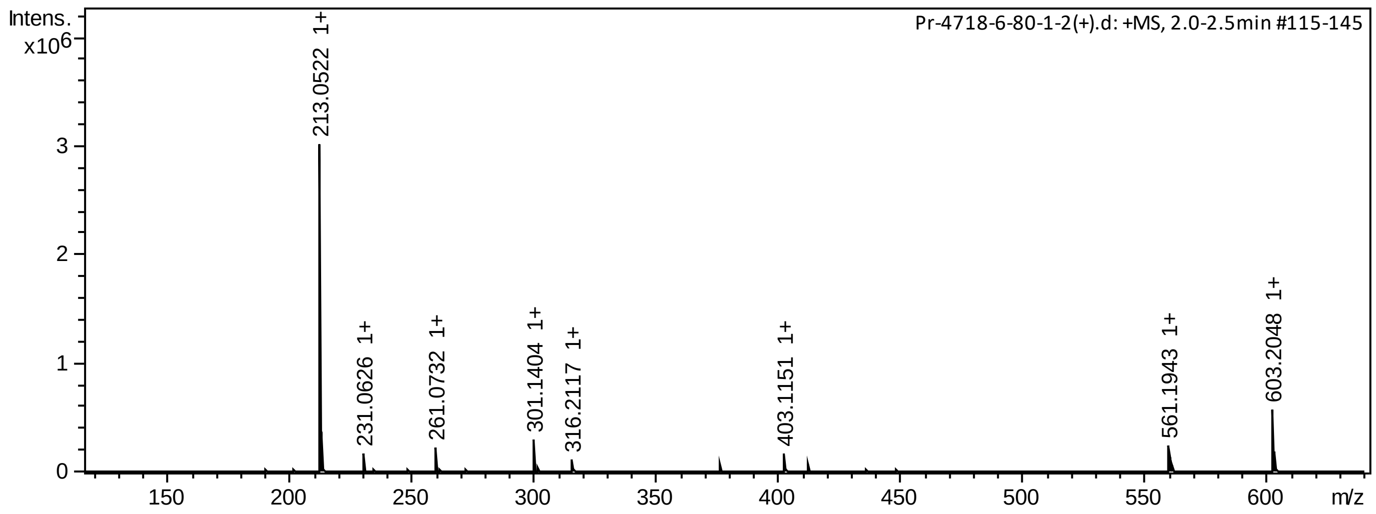
Intens.
x10^6
Pr-4718-6-80-1-2(+).d: +MS, 2.0-2.5min #115-145
213.0522 1+
231.0626 1+
261.0732 1+
301.1404 1+
316.2117 1+
403.1151 1+
561.1943 1+
603.2048 1+
0
1
2
3
150
200
250
300
350
400
450
500
550
600
m/z

Figure S19. ^{1}H **NMR spectrum of 4 measured at 500 MHz in acetone-** d_6

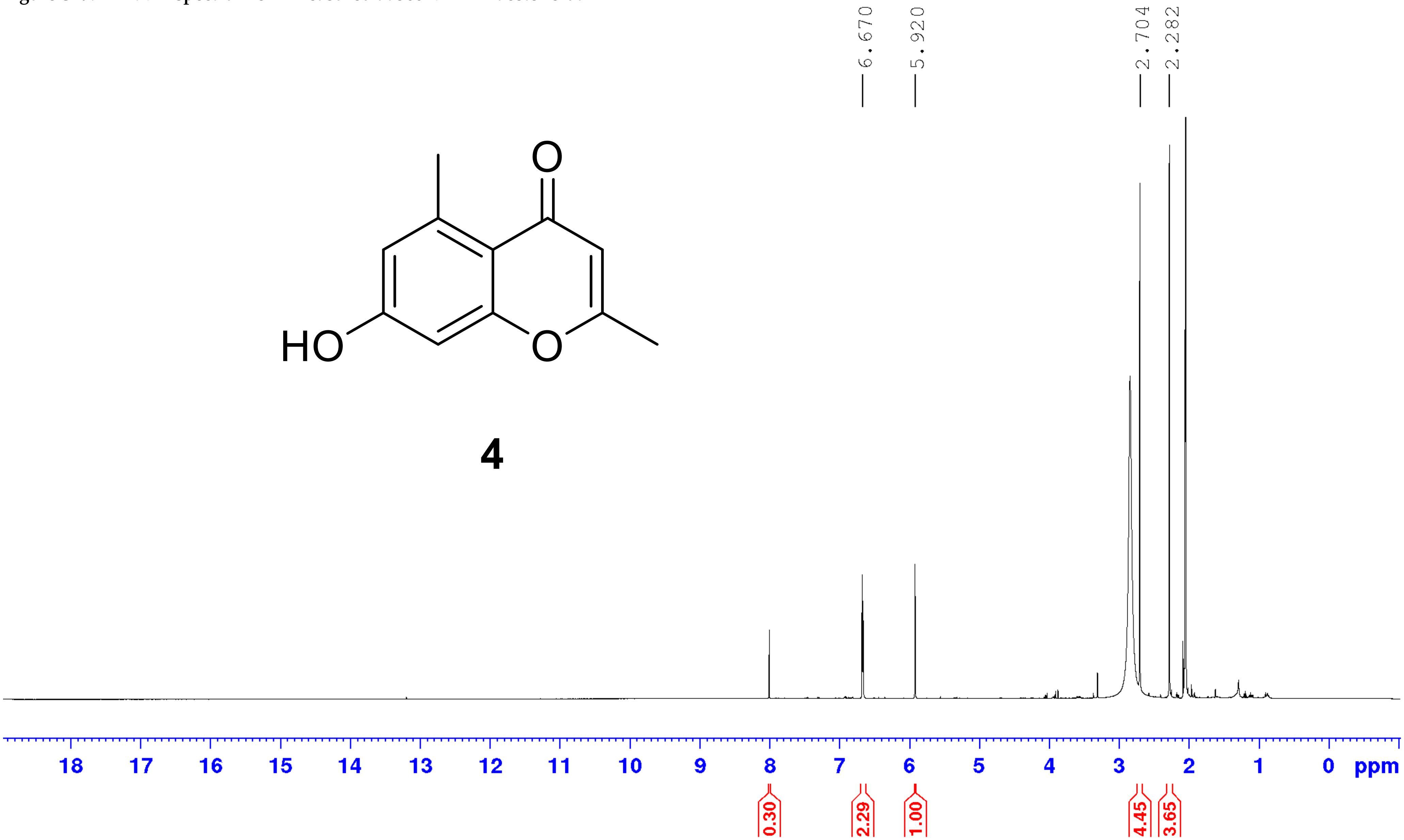

Figure S20. ^{13}C **NMR spectrum of 4 measured at 125 MHz in acetone-d_6**

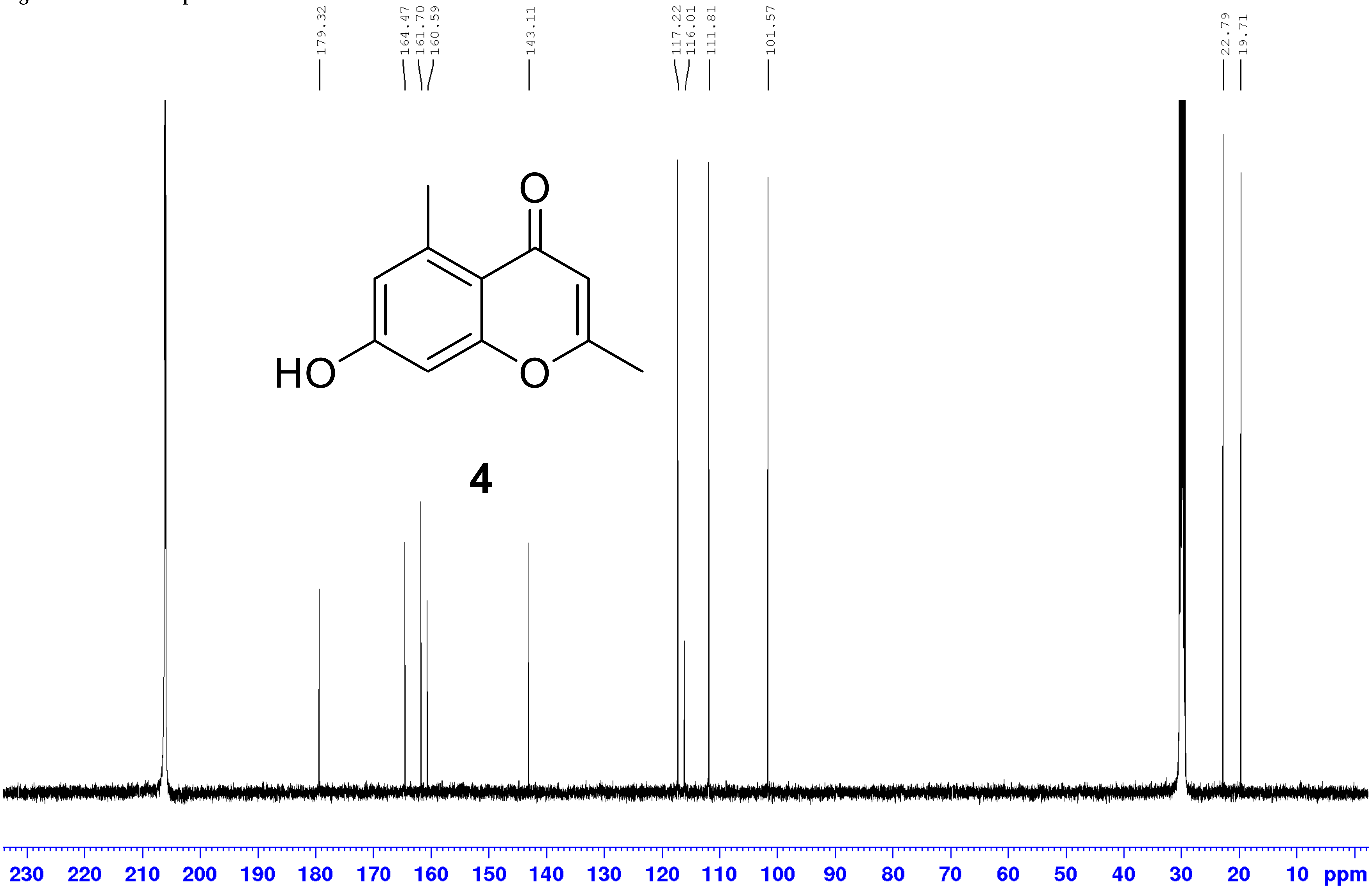

Figure S21. HRESIMS for 5

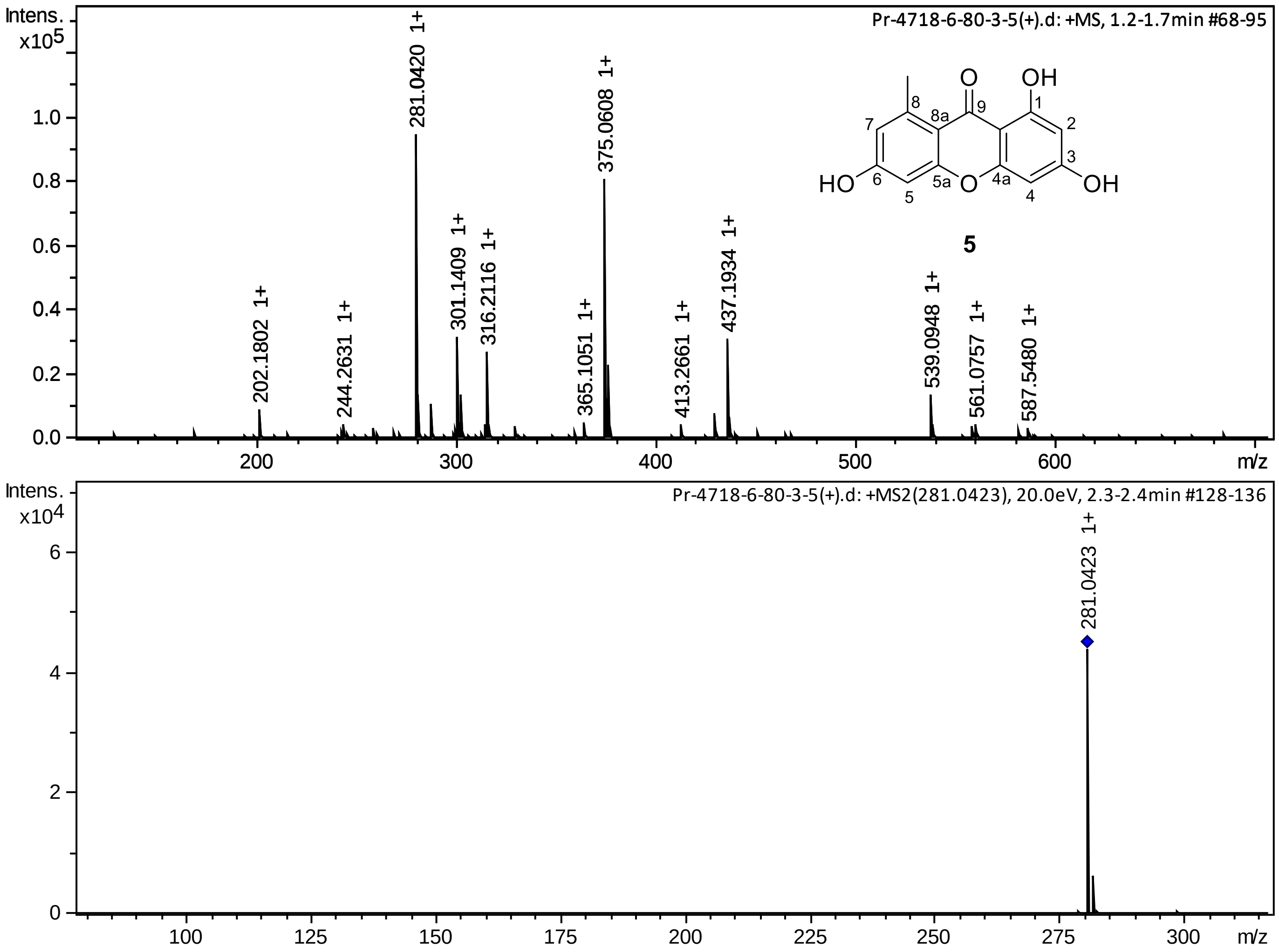

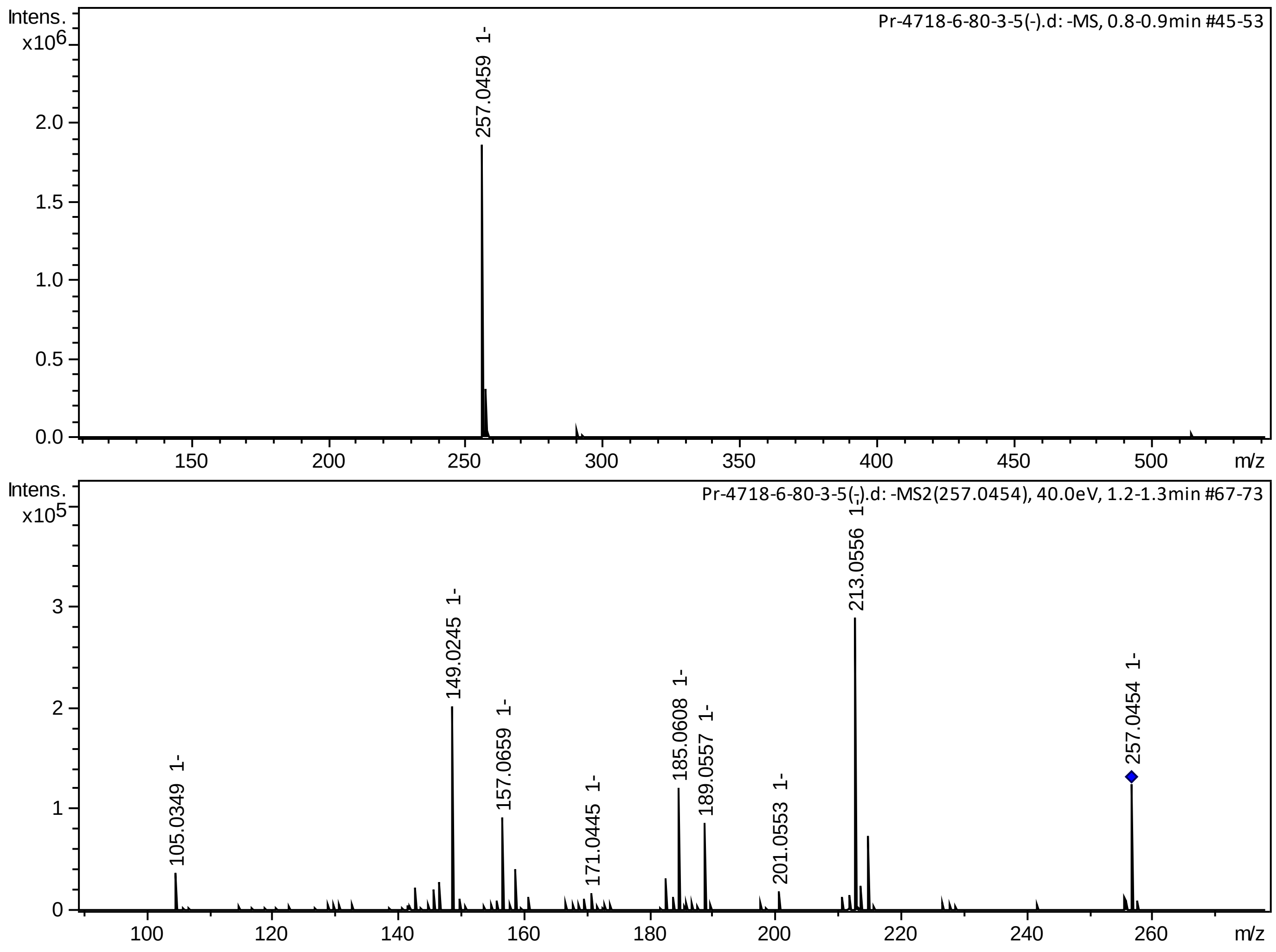

Intens.
x10^6
Pr-4718-6-80-3-5(-).d: -MS, 0.8-0.9min #45-53
257.0459 1-
2.0
1.5
1.0
0.5
0.0
150
200
250
300
350
400
450
500
m/z
Intens.
x10^5
Pr-4718-6-80-3-5(-).d: -MS2(257.0454), 40.0eV, 1.2-1.3min #67-73
213.0556 1-
149.0245 1-
157.0659 1-
185.0608 1-
189.0557 1-
257.0454 1-
105.0349 1-
171.0445 1-
201.0553 1-
3
2
1
0
100
120
140
160
180
200
220
240
260
m/z

Figure S22. ^{1}**H NMR spectrum of 5 measured at 500 MHz in acetone-d$_6$**

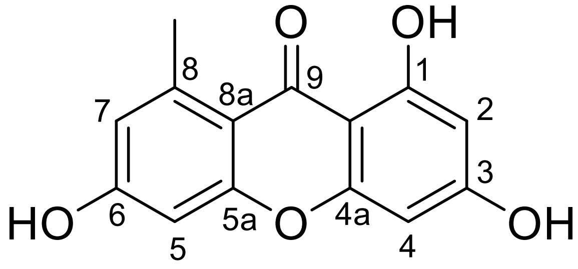

5

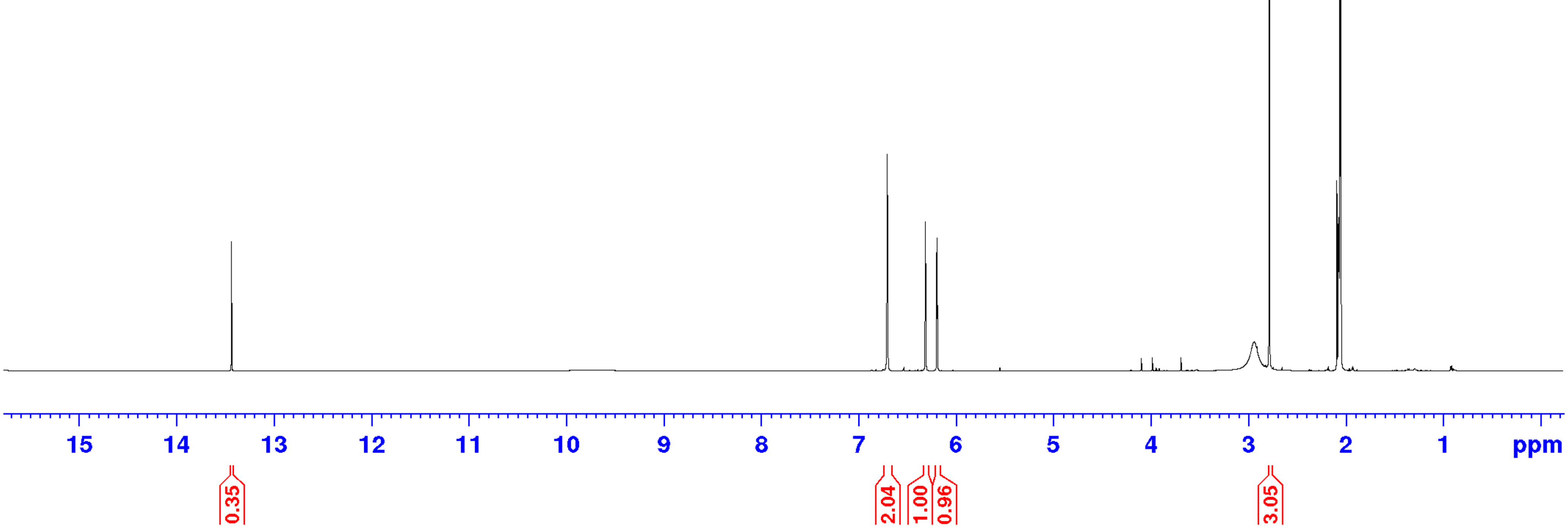

Figure S23. ^{13}C **NMR spectrum of 5 measured at 125 MHz in acetone-d$_6$**

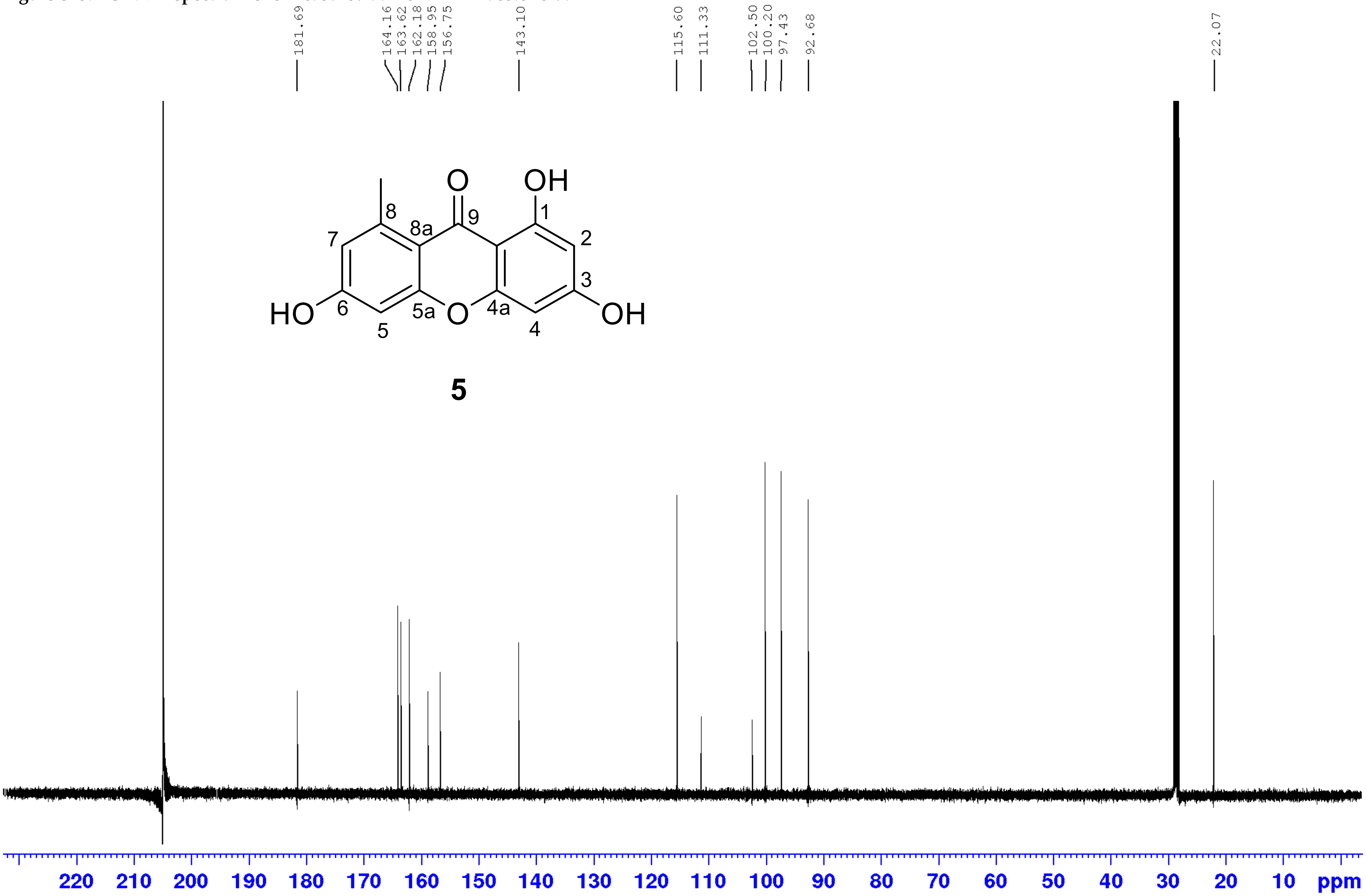

Figure S24. HRESIMS for 6

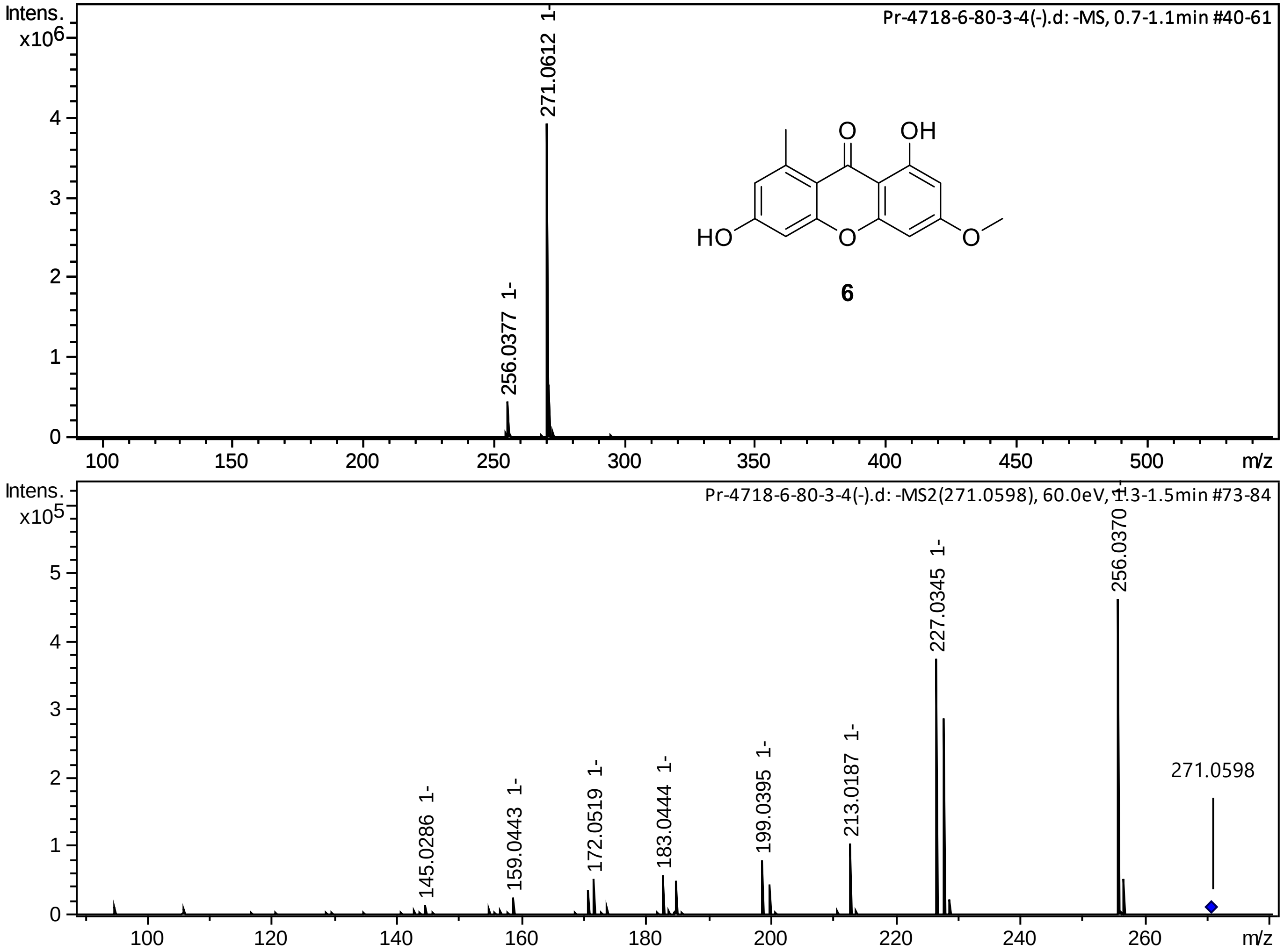

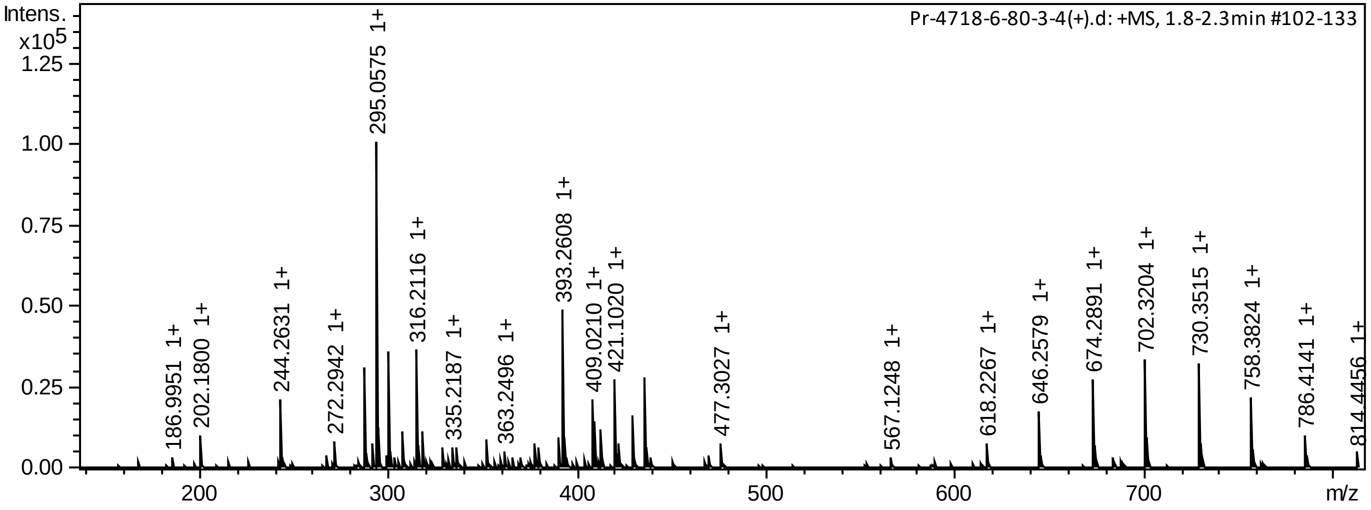

Pr-4718-6-80-3-4(+).d: +MS, 1.8-2.3min #102-133
Intens.
x10^5
m/z
295.0575 1+
393.2608 1+
316.2116 1+
409.0210 1+
421.1020 1+
244.2631 1+
202.1800 1+
186.9951 1+
272.2942 1+
335.2187 1+
363.2496 1+
477.3027 1+
567.1248 1+
618.2267 1+
646.2579 1+
674.2891 1+
702.3204 1+
730.3515 1+
758.3824 1+
786.4141 1+
814.4456 1+

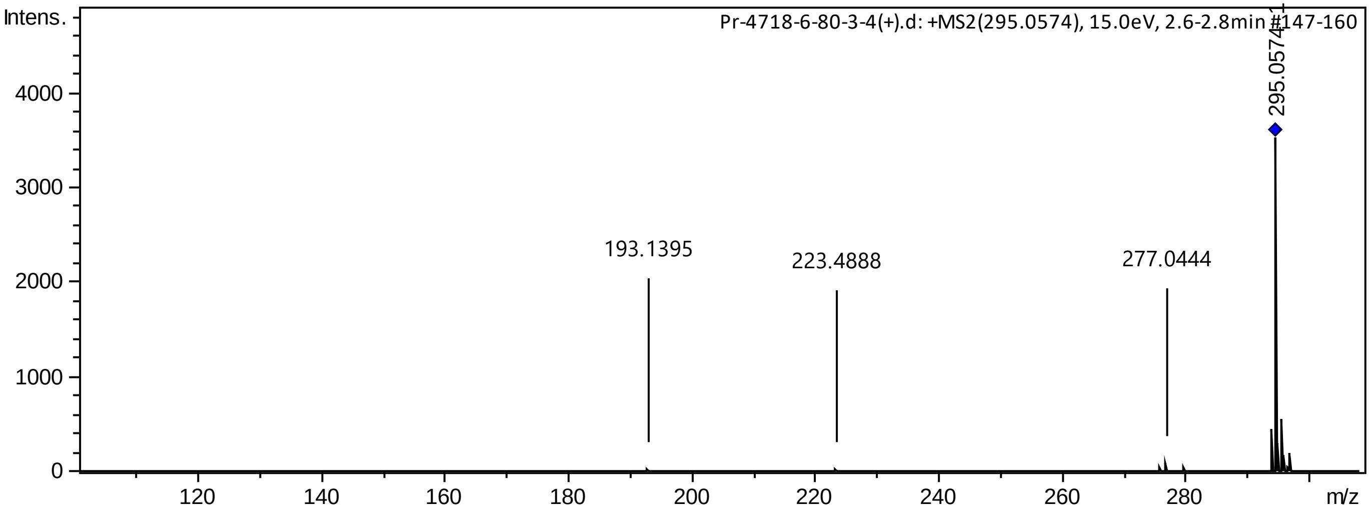

Pr-4718-6-80-3-4(+).d: +MS2(295.0574), 15.0eV, 2.6-2.8min #147-160
Intens.
m/z
295.0574
193.1395
223.4888
277.0444

Figure S25. ^{1}H NMR spectrum of 6 measured at 300 MHz in acetone-d_6

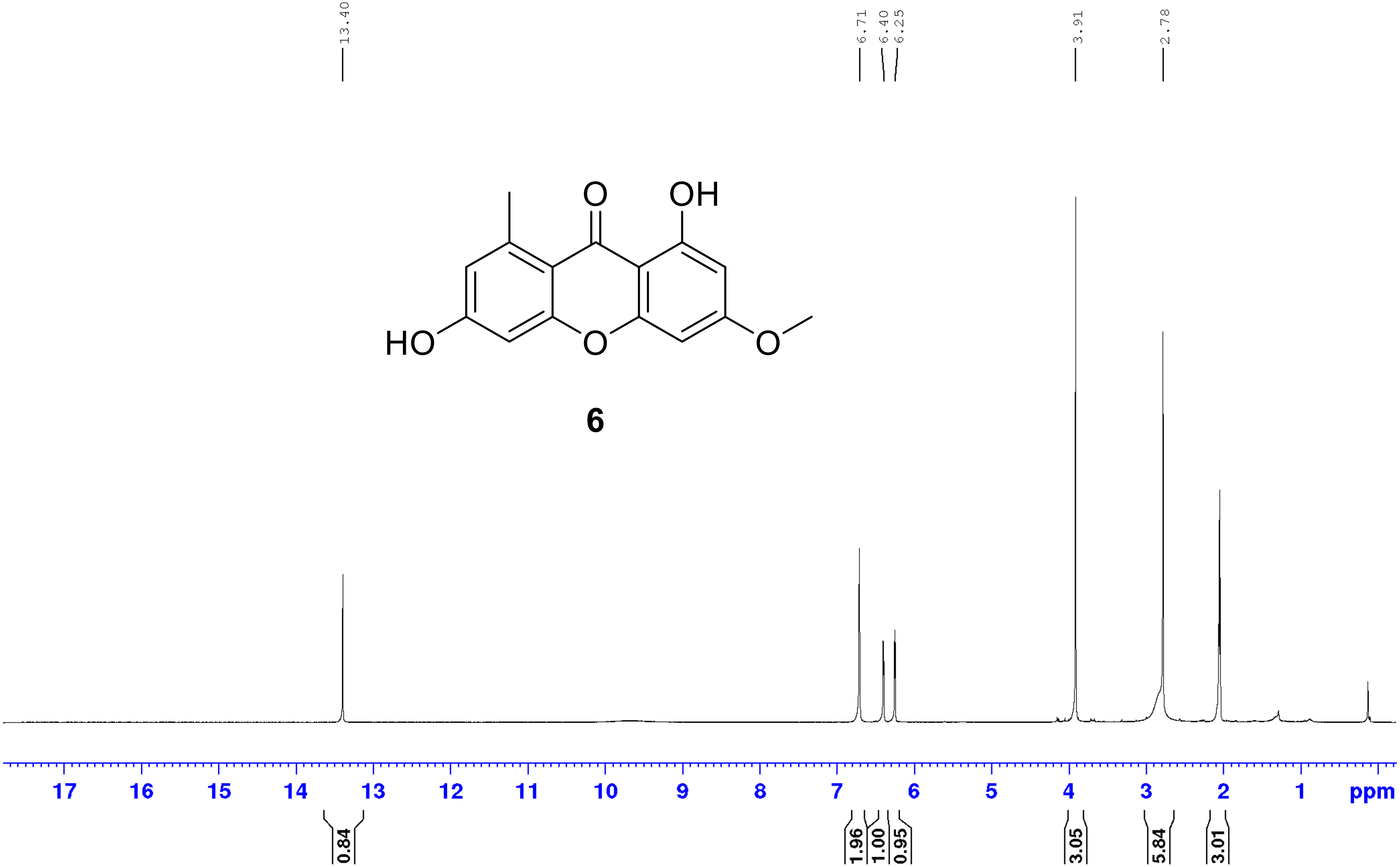

Figure S26. ^{13}C **NMR spectrum of 6 measured at 75 MHz in acetone-d**$_6$

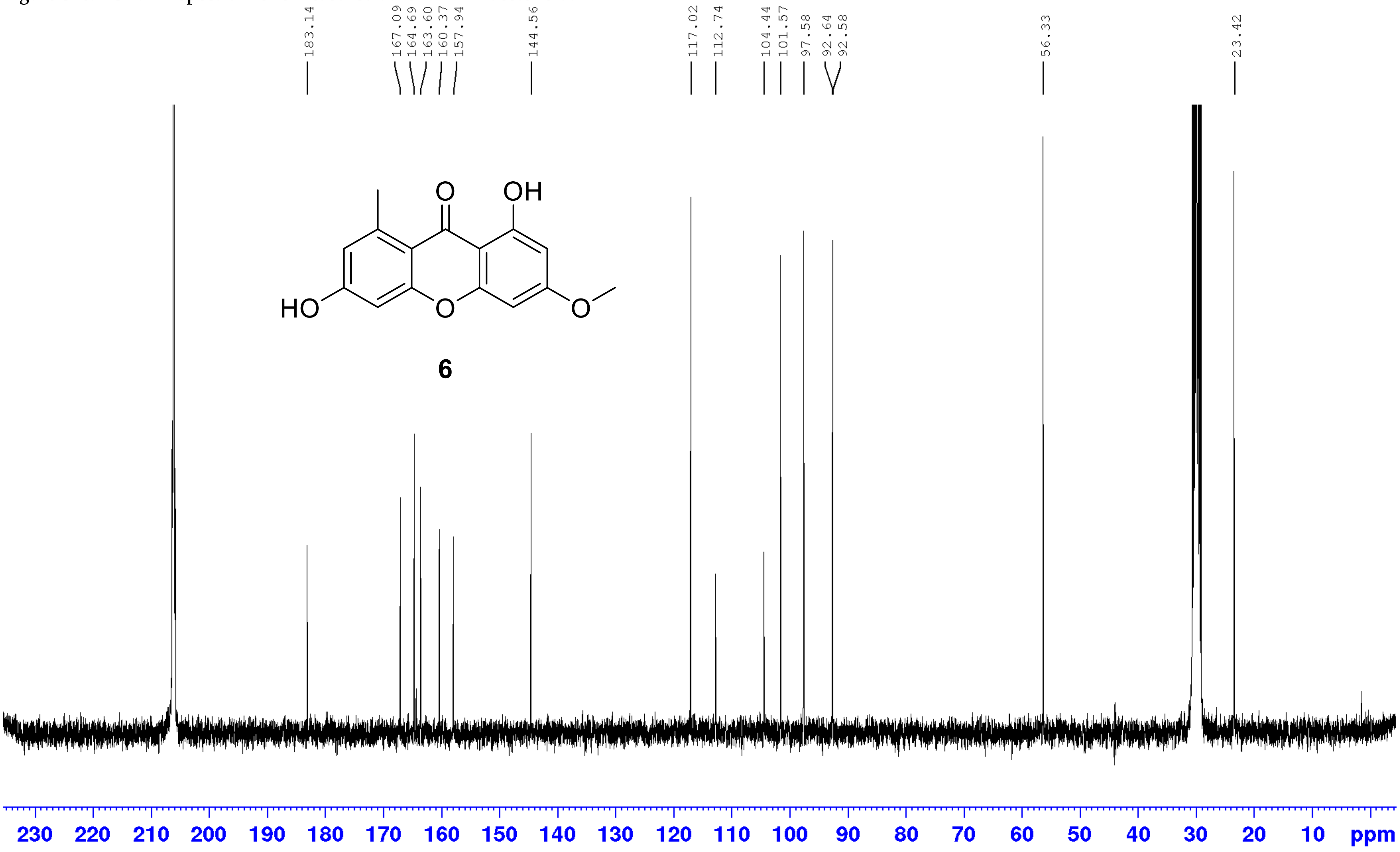

Figure S27. HRESIMS for 7

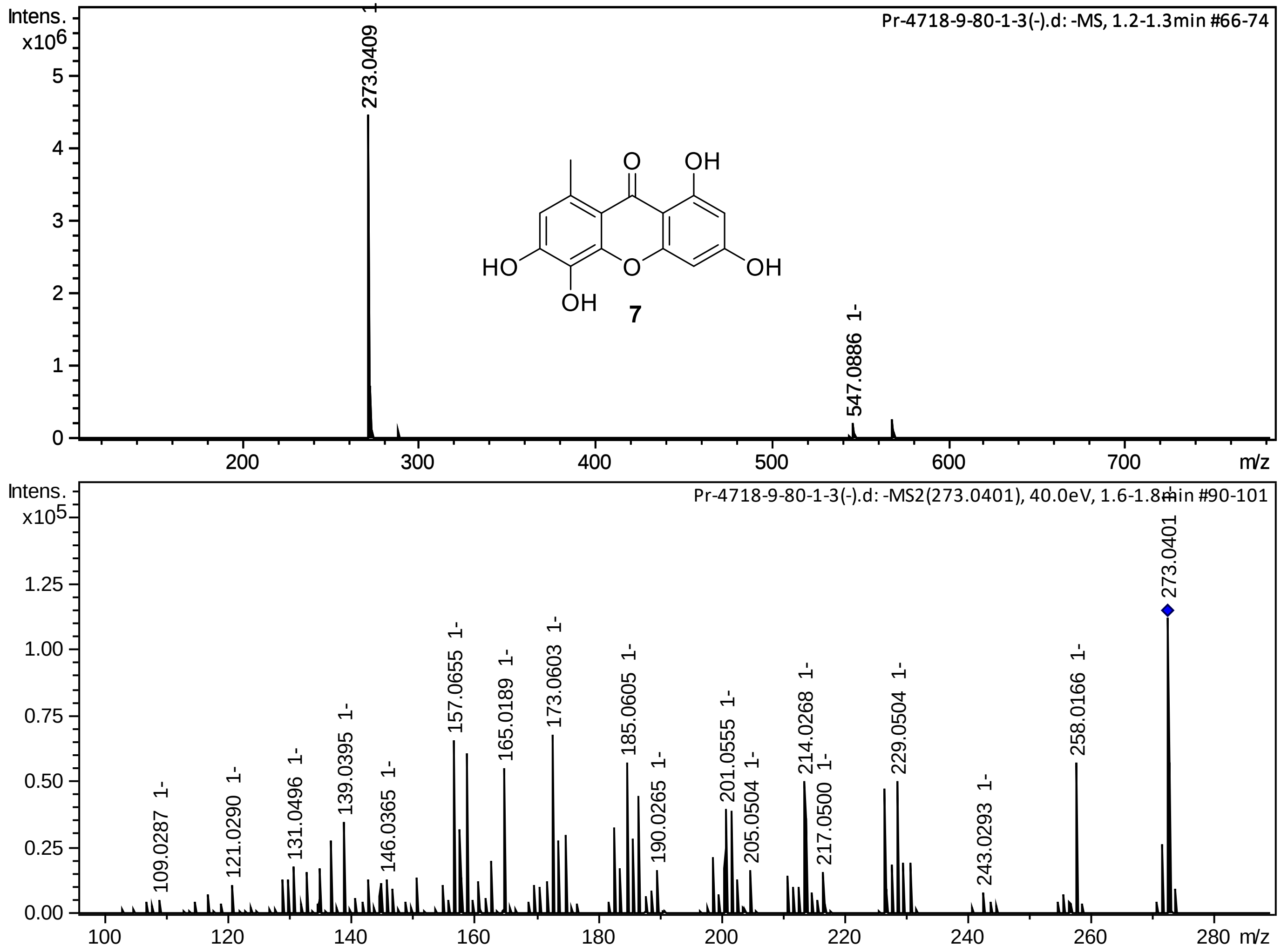

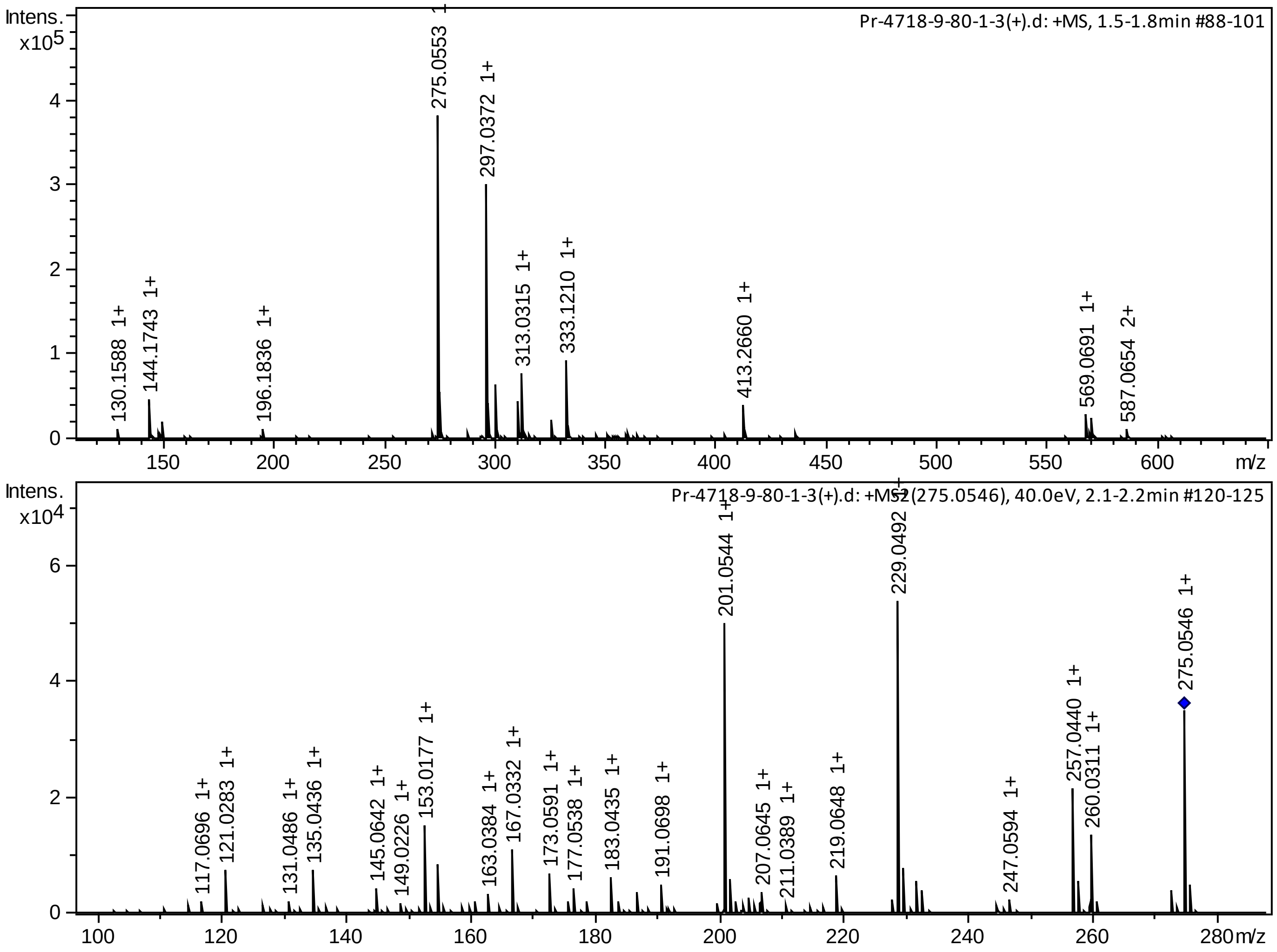

Pr-4718-9-80-1-3(+).d: +MS, 1.5-1.8min #88-101
Intens. x10^5
275.0553
297.0372 1+
130.1588 1+
144.1743 1+
196.1836 1+
313.0315 1+
333.1210 1+
413.2660 1+
569.0691 1+
587.0654 2+
m/z
Pr-4718-9-80-1-3(+).d: +MS2(275.0546), 40.0eV, 2.1-2.2min #120-125
Intens. x10^4
117.0696 1+
121.0283 1+
131.0486 1+
135.0436 1+
145.0642 1+
149.0226 1+
153.0177 1+
163.0384 1+
167.0332 1+
173.0591 1+
177.0538 1+
183.0435 1+
191.0698 1+
201.0544 1+
207.0645 1+
211.0389 1+
219.0648 1+
229.0492
247.0594 1+
257.0440 1+
260.0311 1+
275.0546 1+
m/z

Figure S28. ^1H **NMR spectrum of 7 measured at 700 MHz in acetone-d_6**

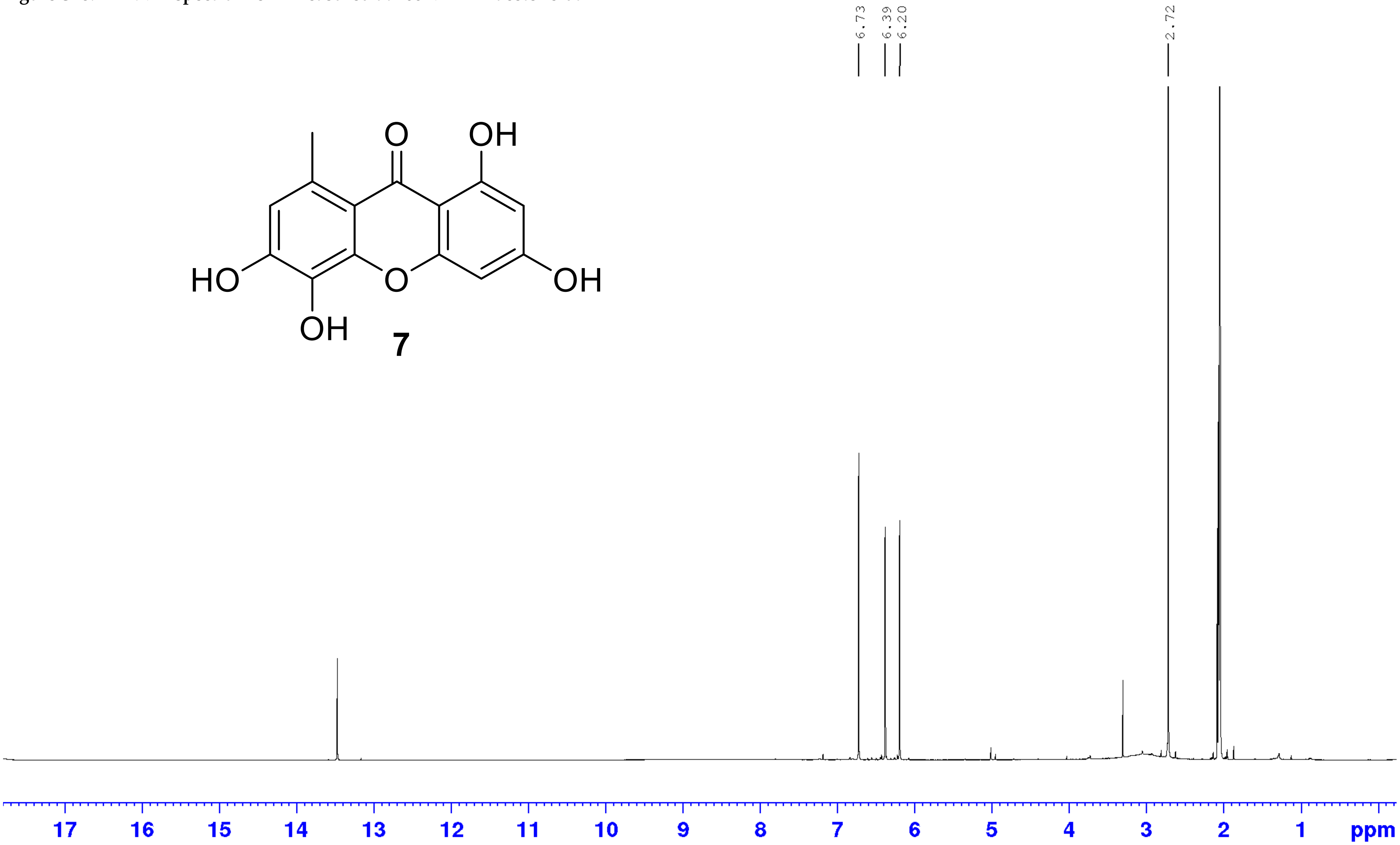

Figure S29. ^{13}C NMR spectrum of 7 measured at 175 MHz in acetone-d_6

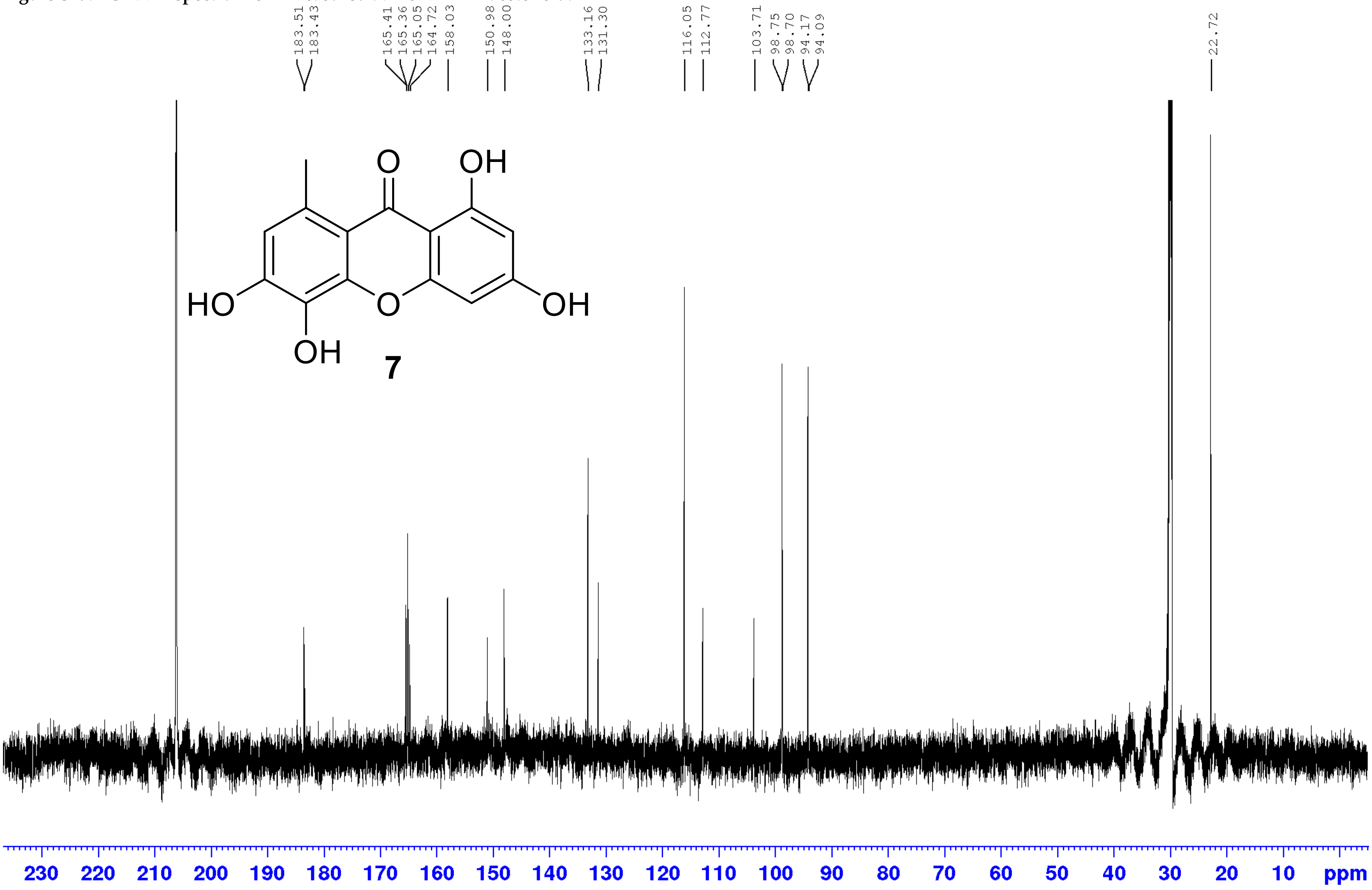

Figure S30. HRESIMS for 8

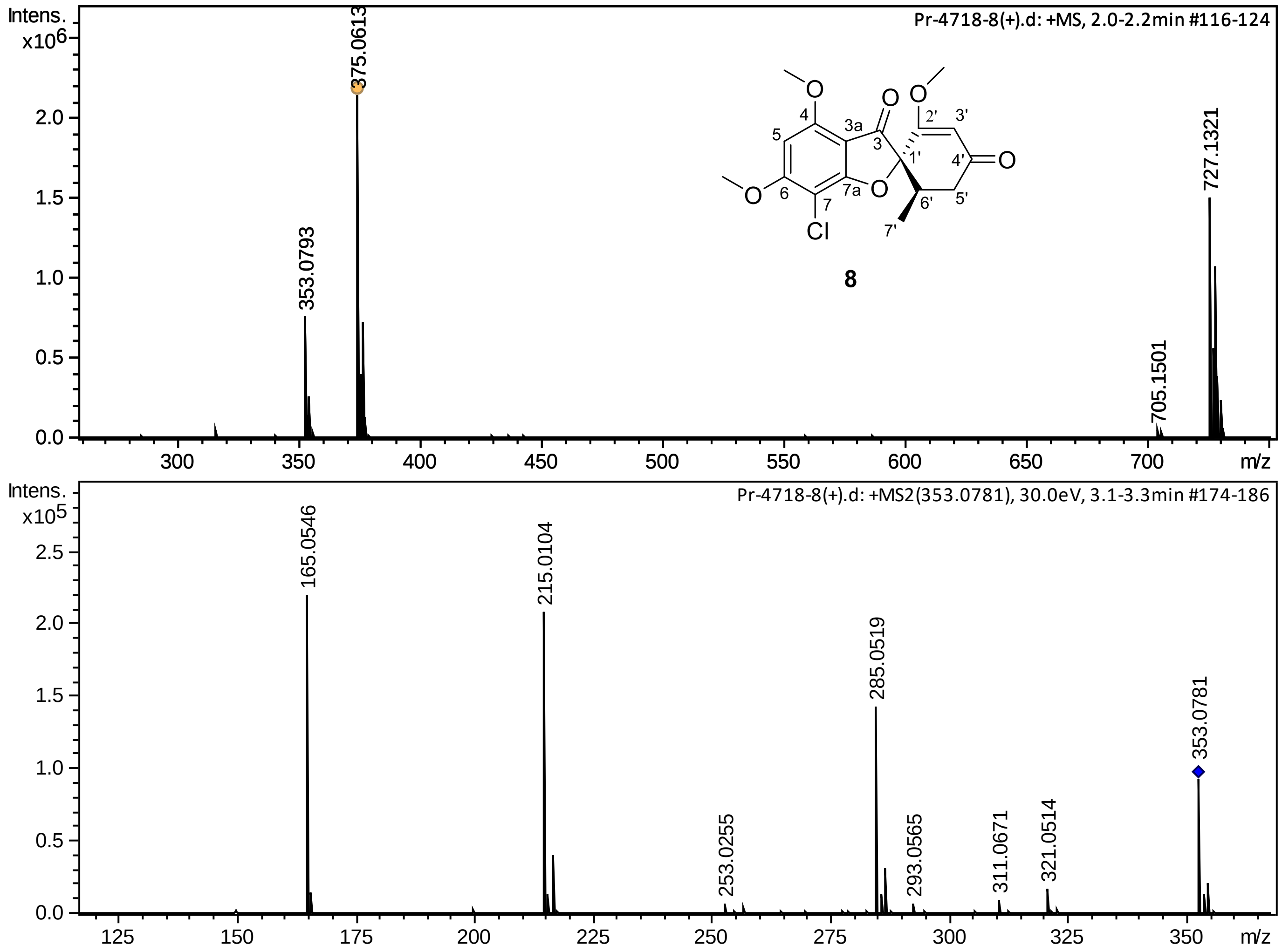

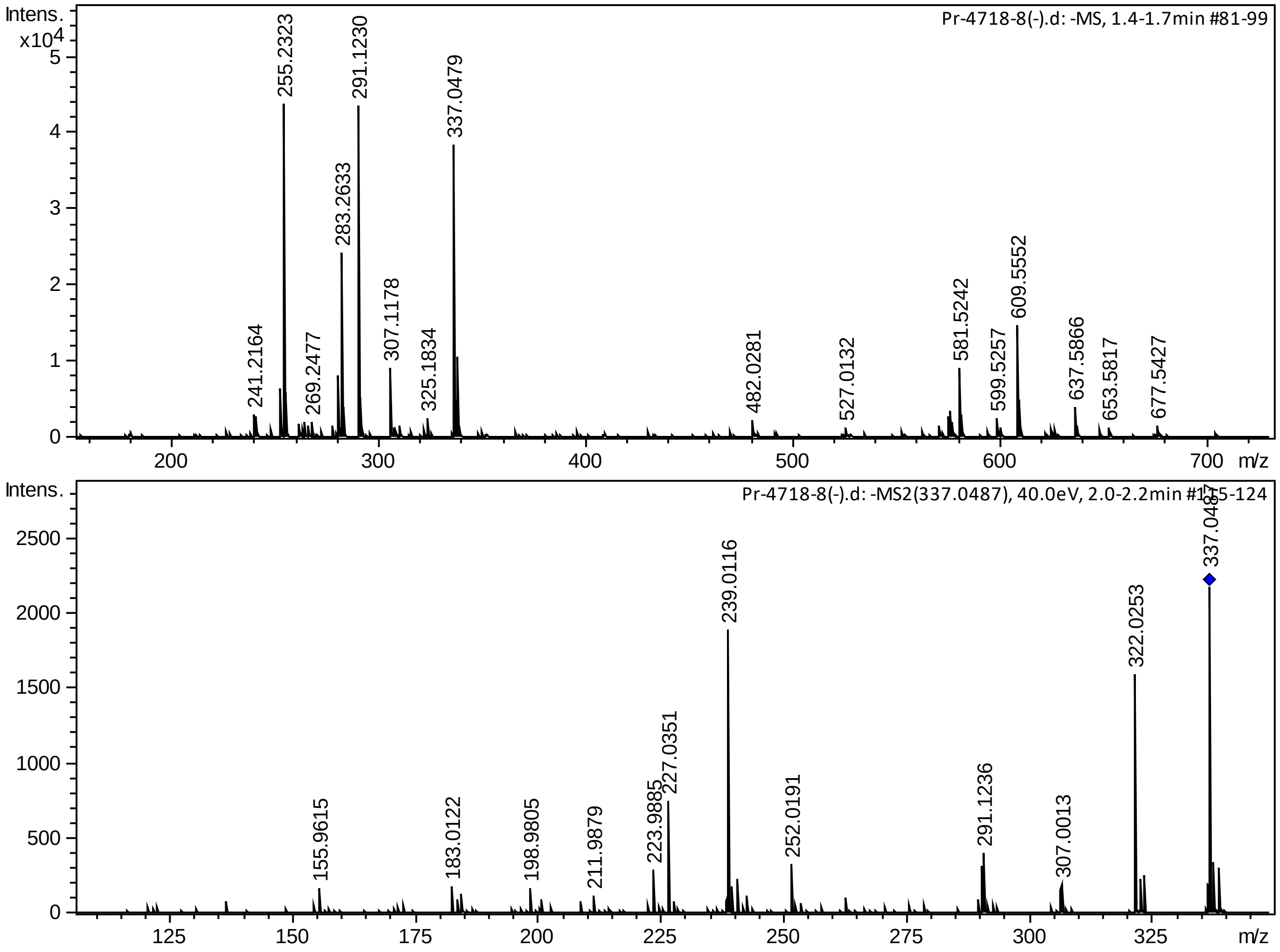

Intens.
x10^4
Pr-4718-8(-).d: -MS, 1.4-1.7min #81-99
255.2323
291.1230
337.0479
283.2633
307.1178
241.2164
269.2477
325.1834
482.0281
527.0132
581.5242
599.5257
609.5552
637.5866
653.5817
677.5427
m/z
Intens.
Pr-4718-8(-).d: -MS2(337.0487), 40.0eV, 2.0-2.2min #115-124
337.0487
322.0253
239.0116
227.0351
223.9885
252.0191
291.1236
307.0013
155.9615
183.0122
198.9805
211.9879
m/z

Figure S31. ^{1}H NMR spectrum of 8 measured at 700 MHz in acetone-d_6

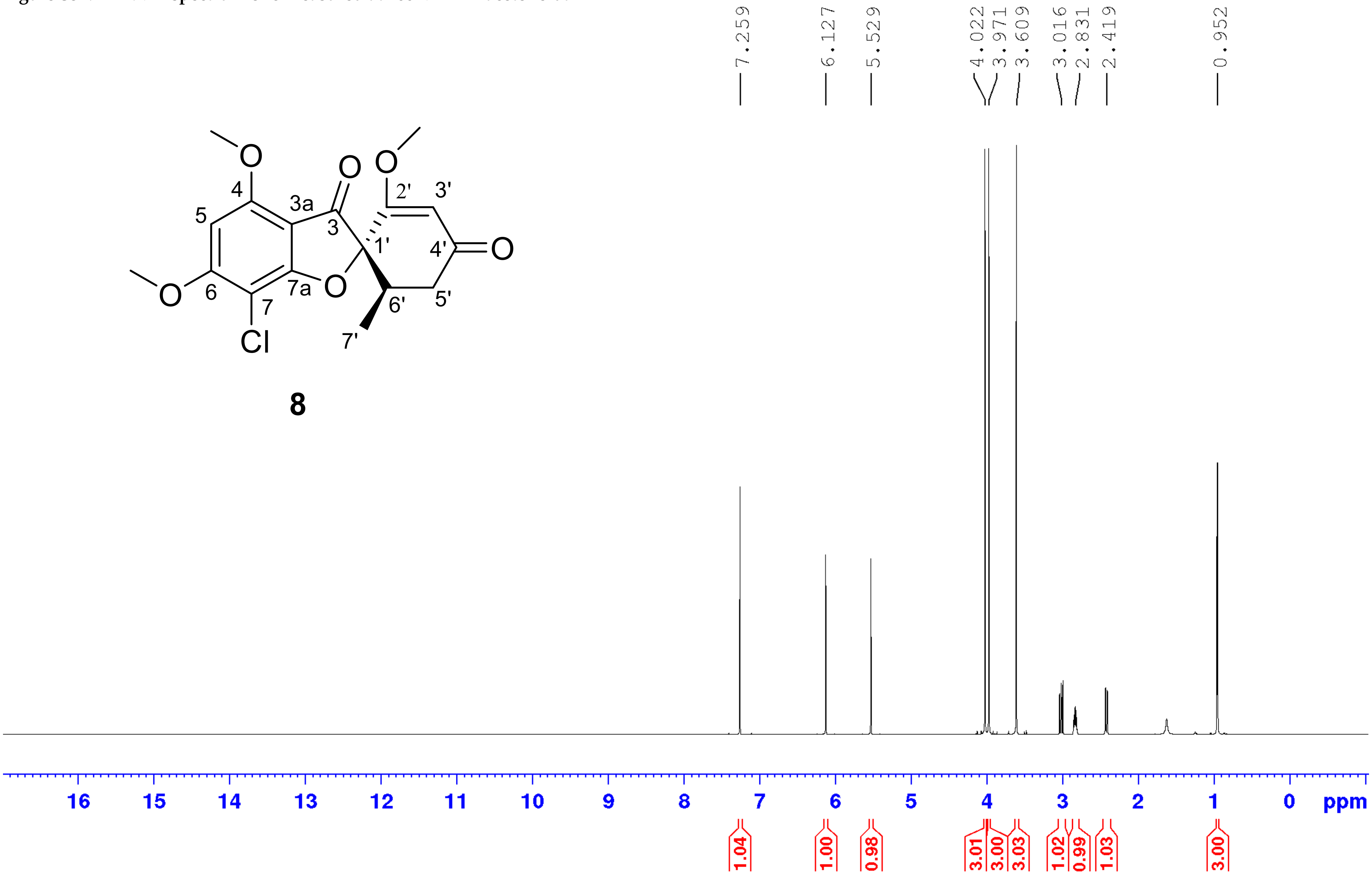

Figure S32. ^{13}C NMR spectrum of 8 measured at 175 MHz in acetone-d$_6$

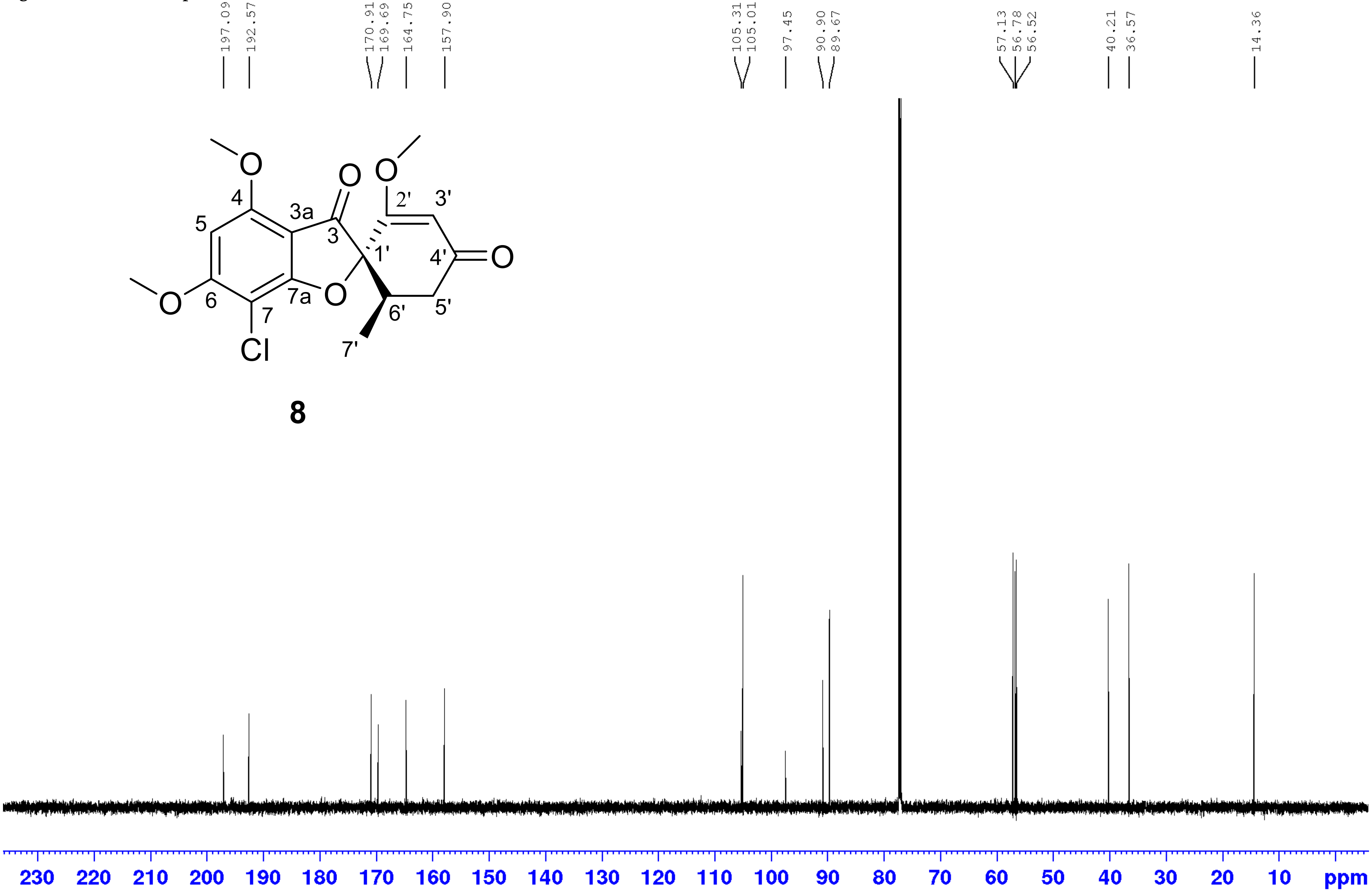

Figure S33. HRESIMS for 9

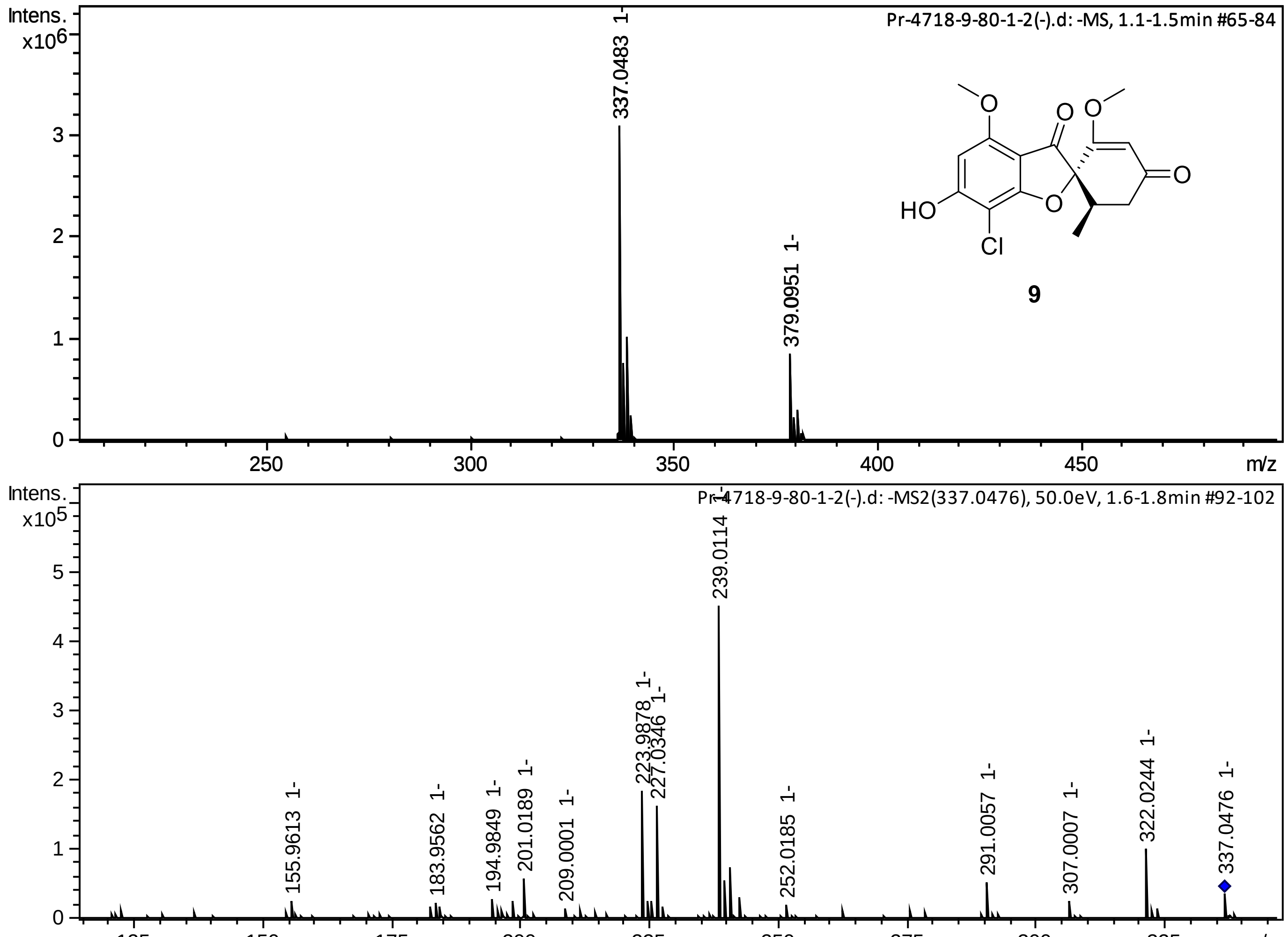

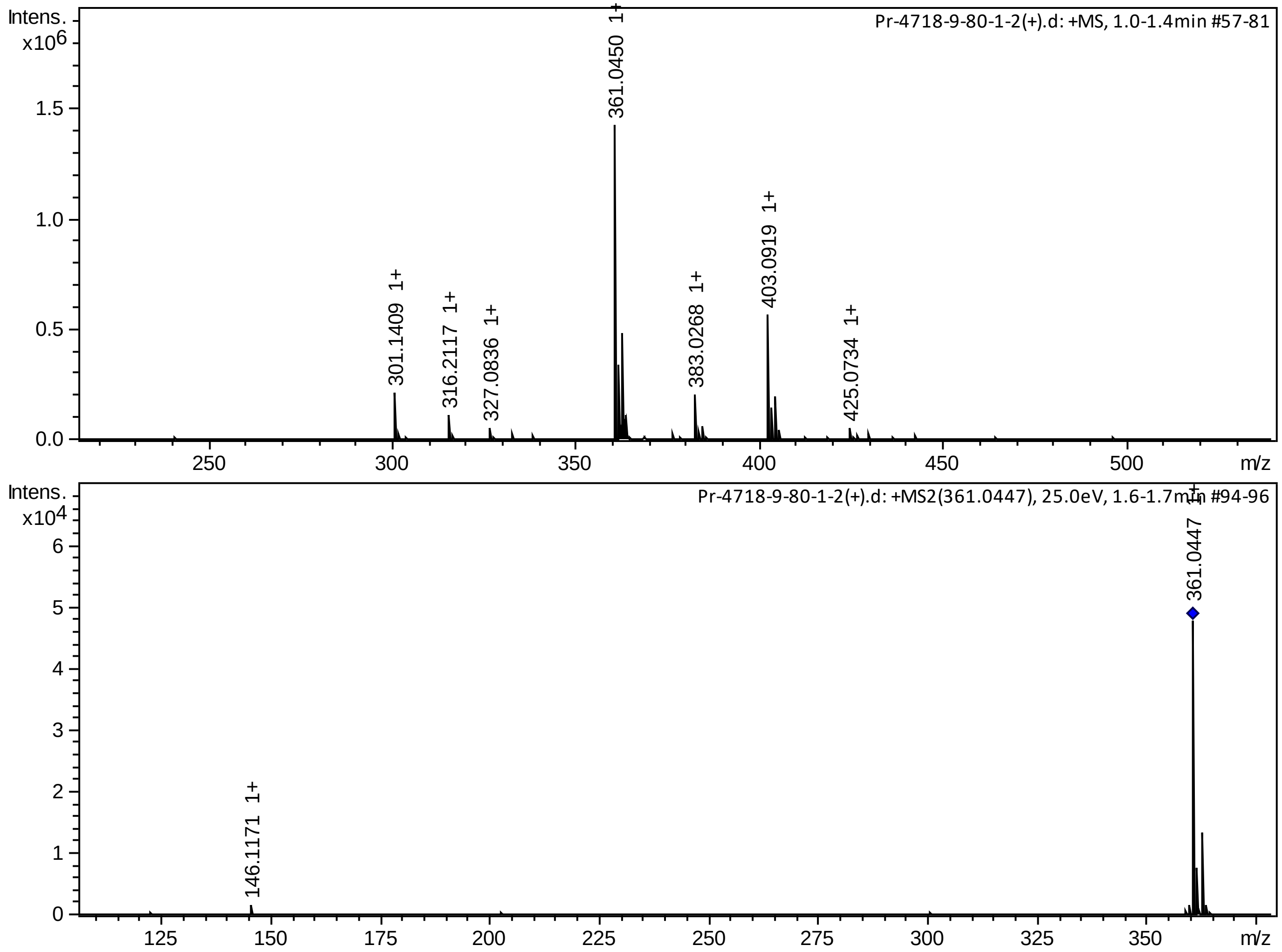

Intens.
x10^6
Pr-4718-9-80-1-2(+).d: +MS, 1.0-1.4min #57-81
361.0450 1+
403.0919 1+
301.1409 1+
316.2117 1+
327.0836 1+
383.0268 1+
425.0734 1+
m/z
Intens.
x10^4
Pr-4718-9-80-1-2(+).d: +MS2(361.0447), 25.0eV, 1.6-1.7min #94-96
361.0447
146.1171 1+
m/z

Figure S34. ^{1}H NMR spectrum of 9 measured at 700 MHz in acetone-d_6

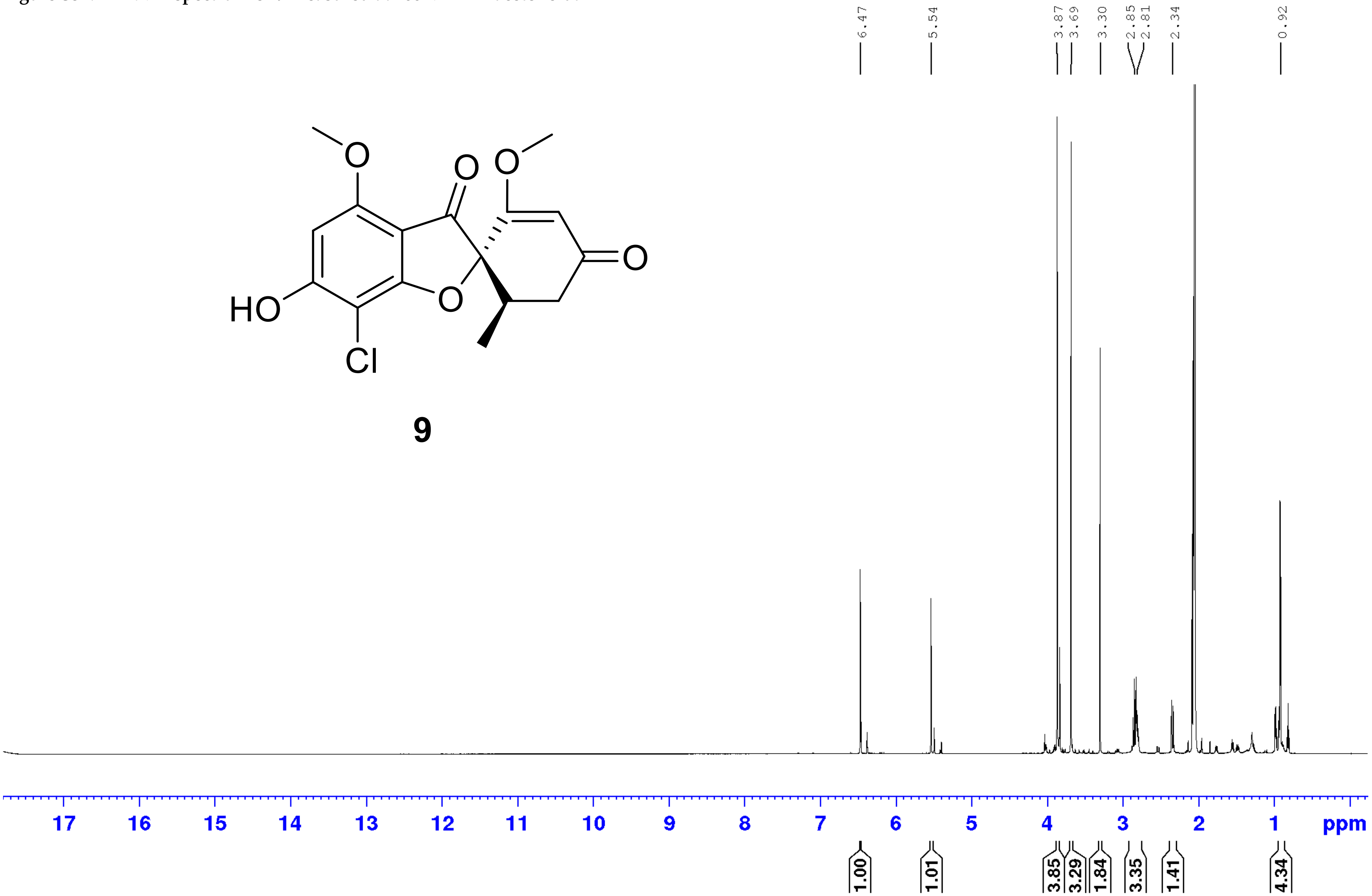

Figure S35. ^{13}C NMR spectrum of **9** measured at 175 MHz in acetone-d_6

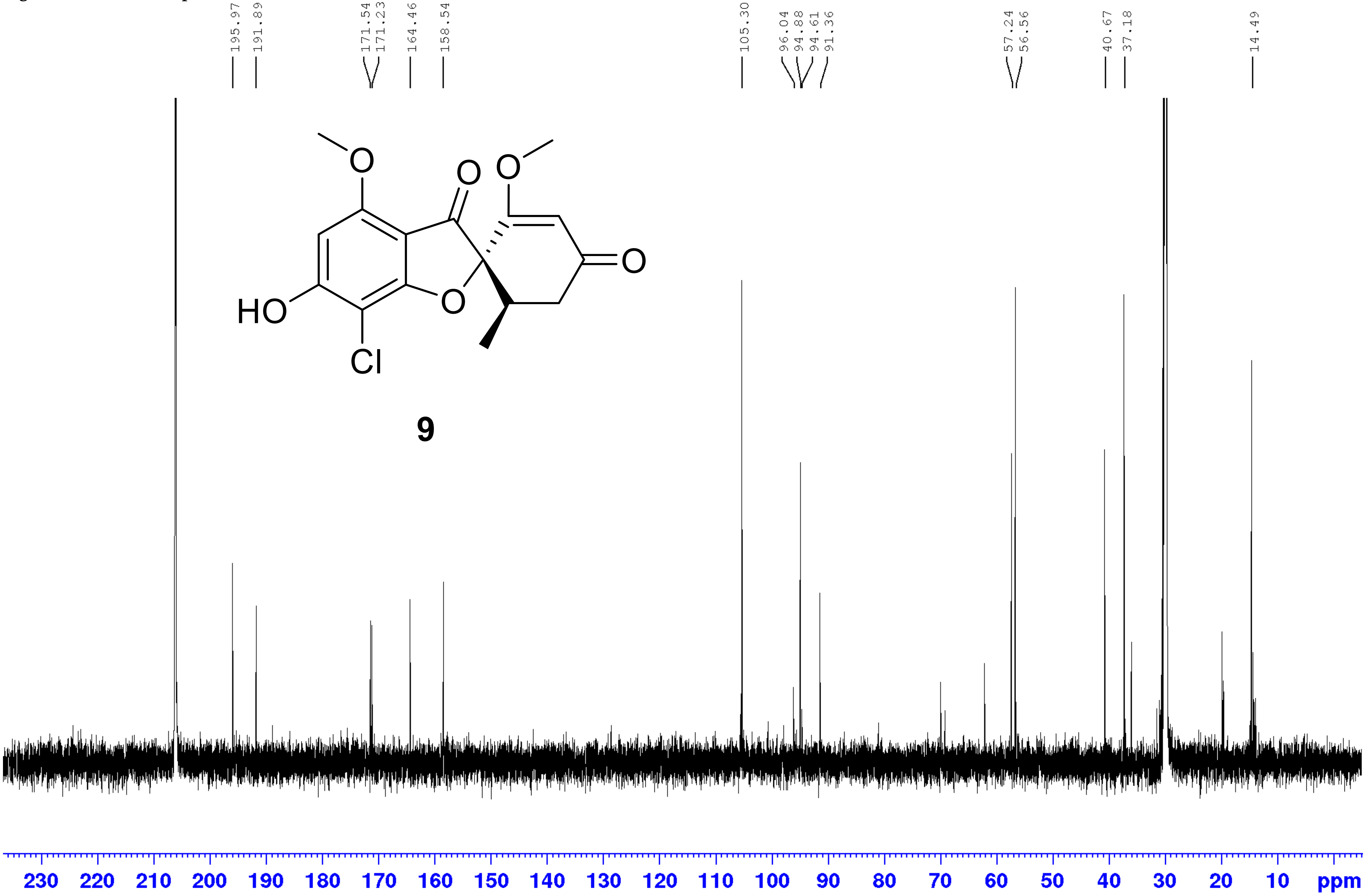

Figure S36. HRESIMS for 10

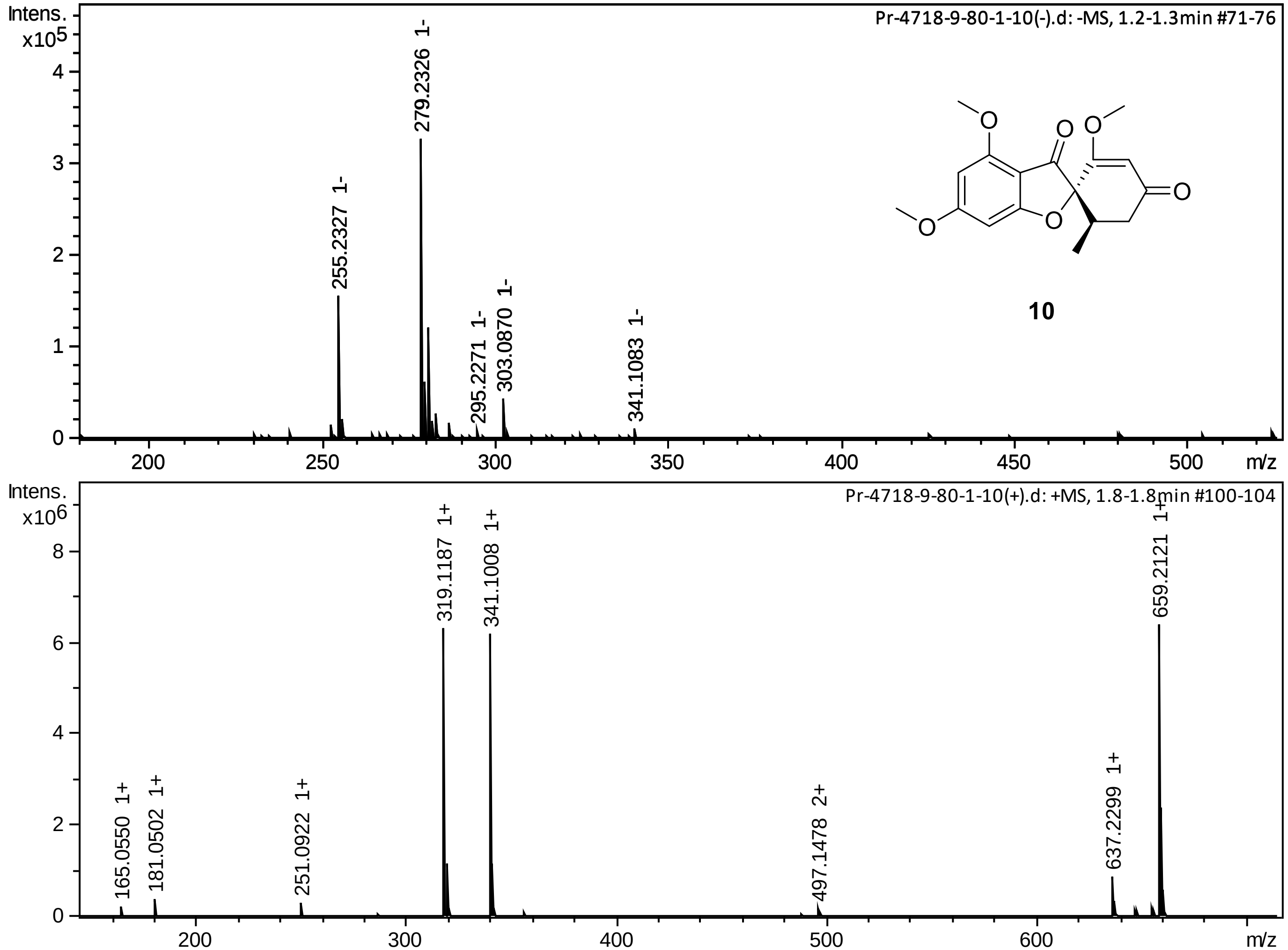

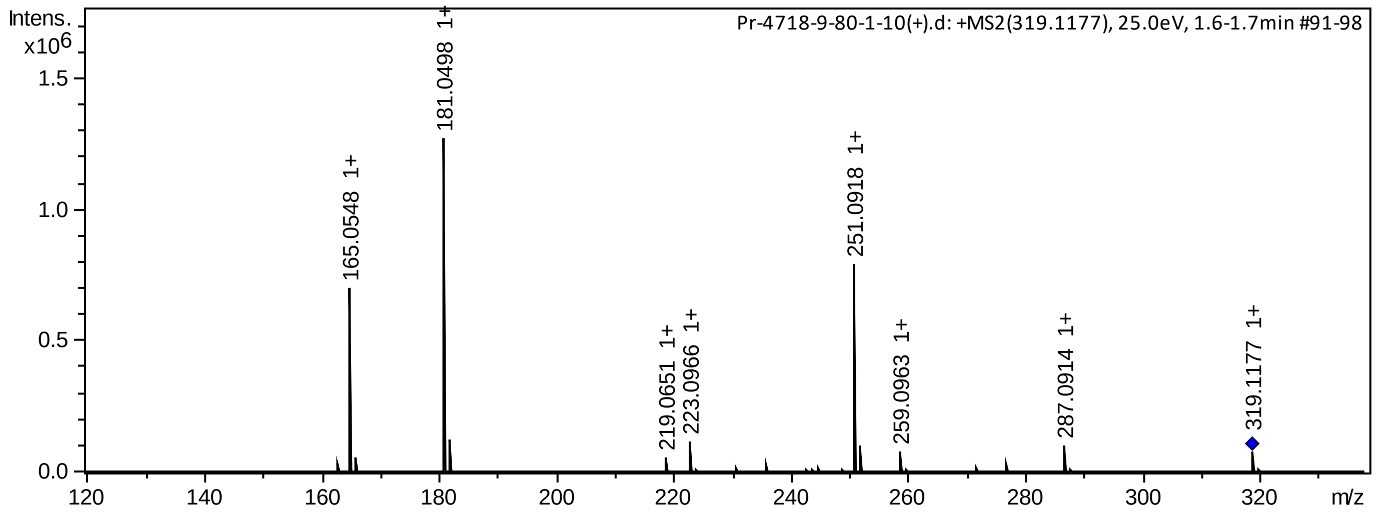

Intens.
x10^6
1.5
1.0
0.5
0.0
Pr-4718-9-80-1-10(+).d: +MS2(319.1177), 25.0eV, 1.6-1.7min #91-98
165.0548 1+
181.0498 1+
219.0651 1+
223.0966 1+
251.0918 1+
259.0963 1+
287.0914 1+
319.1177 1+
120
140
160
180
200
220
240
260
280
300
320
m/z

Figure S37. ^{1}H NMR spectrum of 10 measured at 500 MHz in acetone-d_6

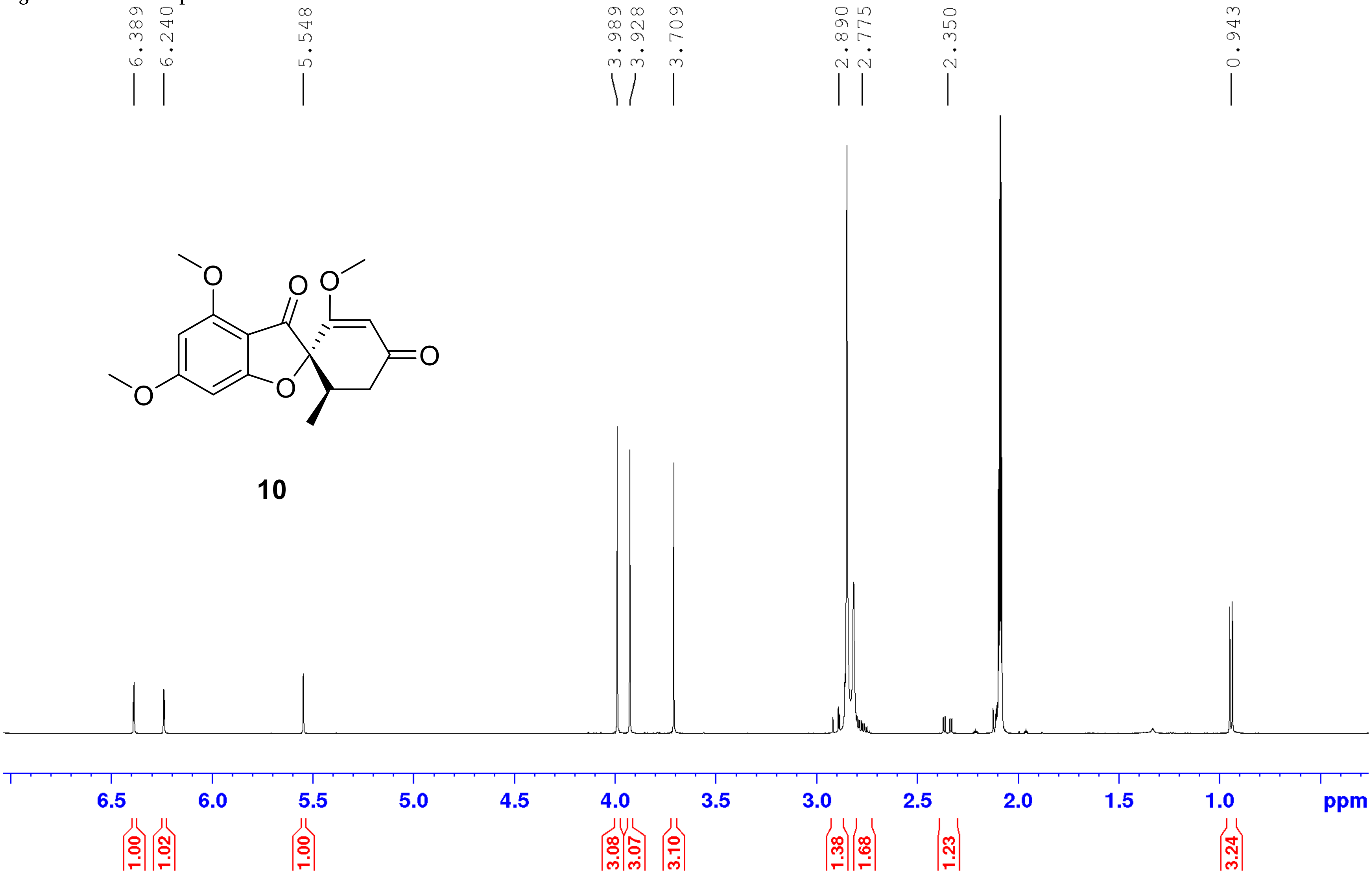

Figure S38. ^{13}C **NMR spectrum of 10 measured at 125 MHz in acetone-**d_6

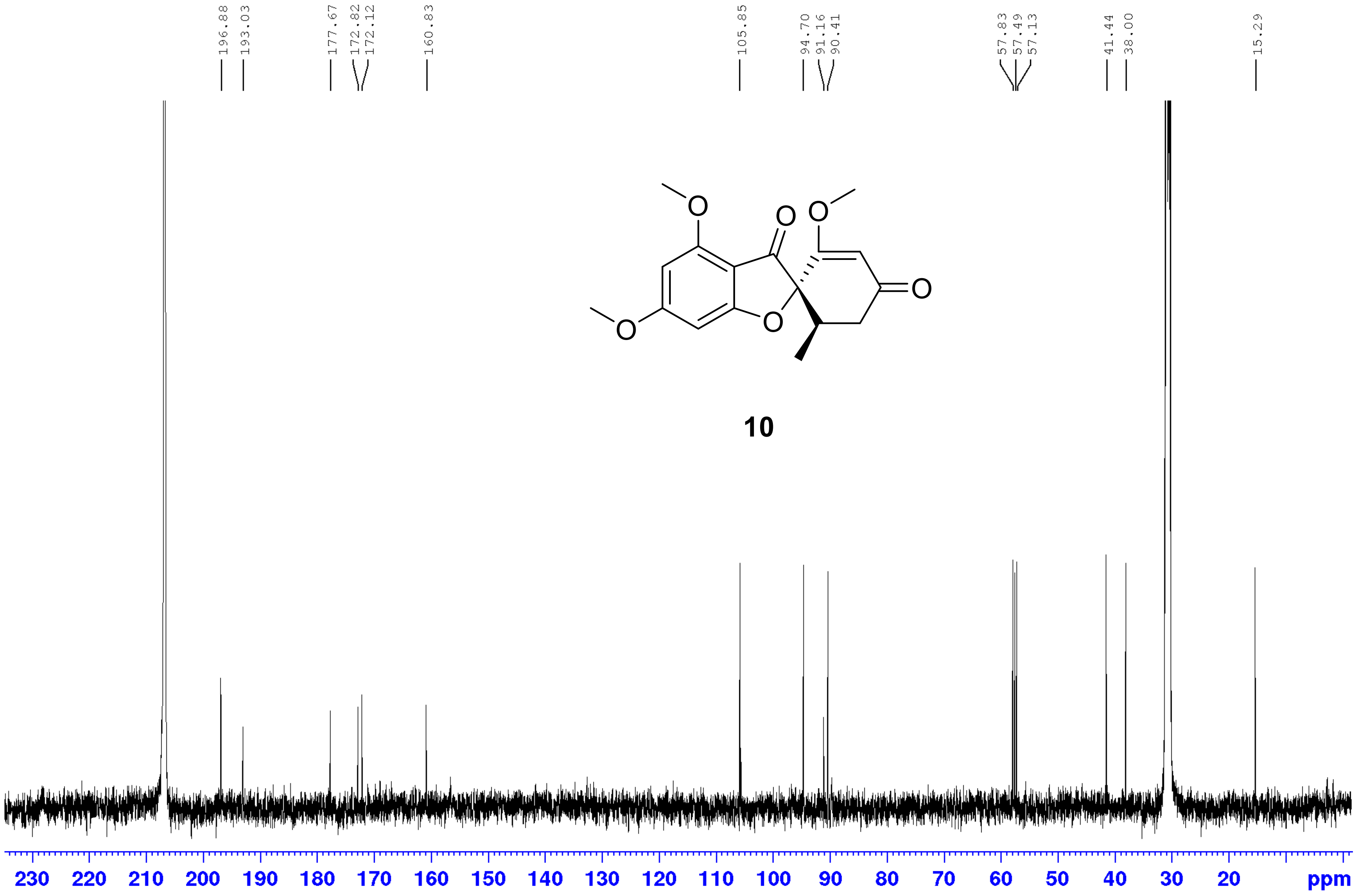

Figure S39. HRESIMS for 11

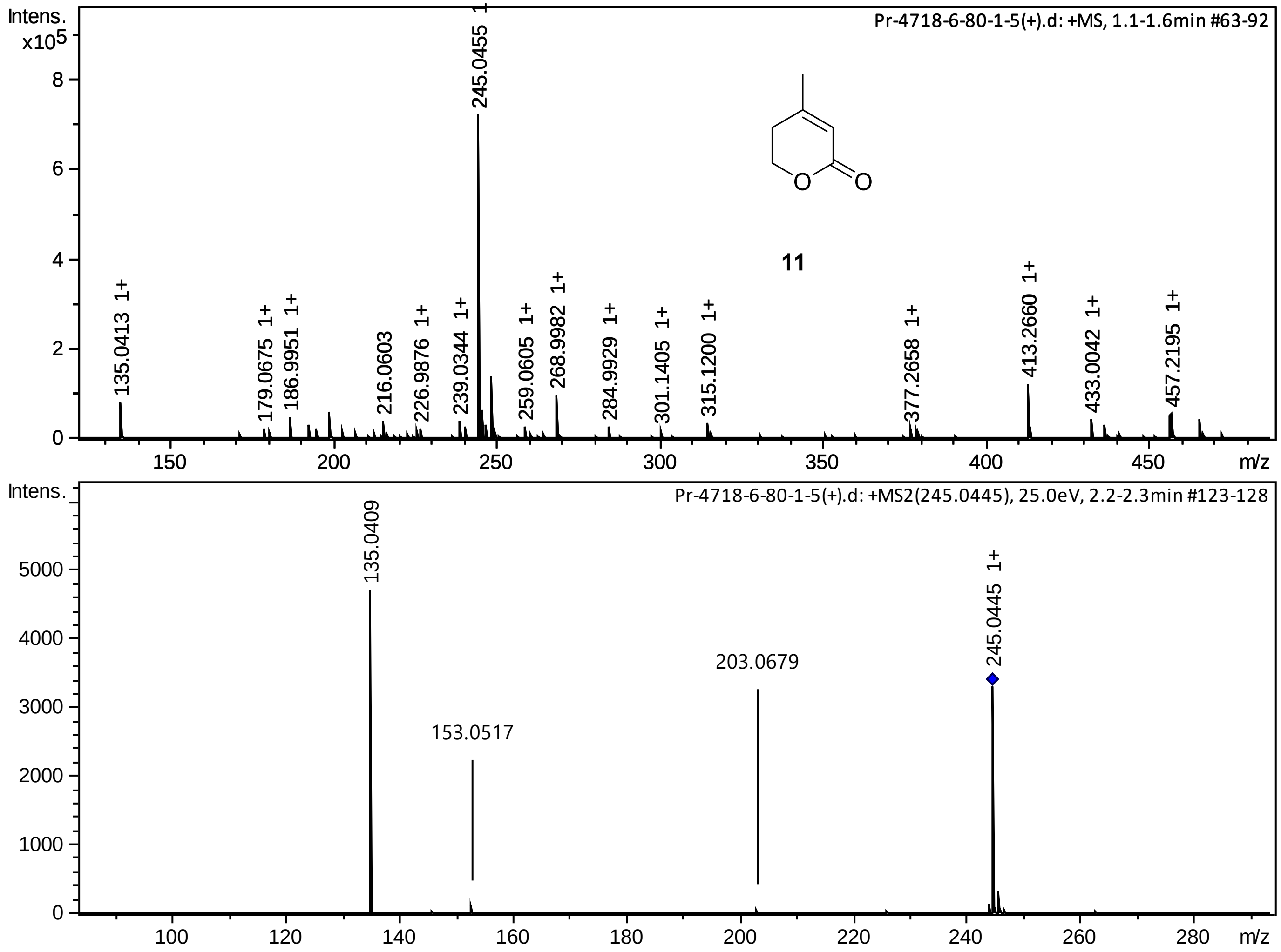

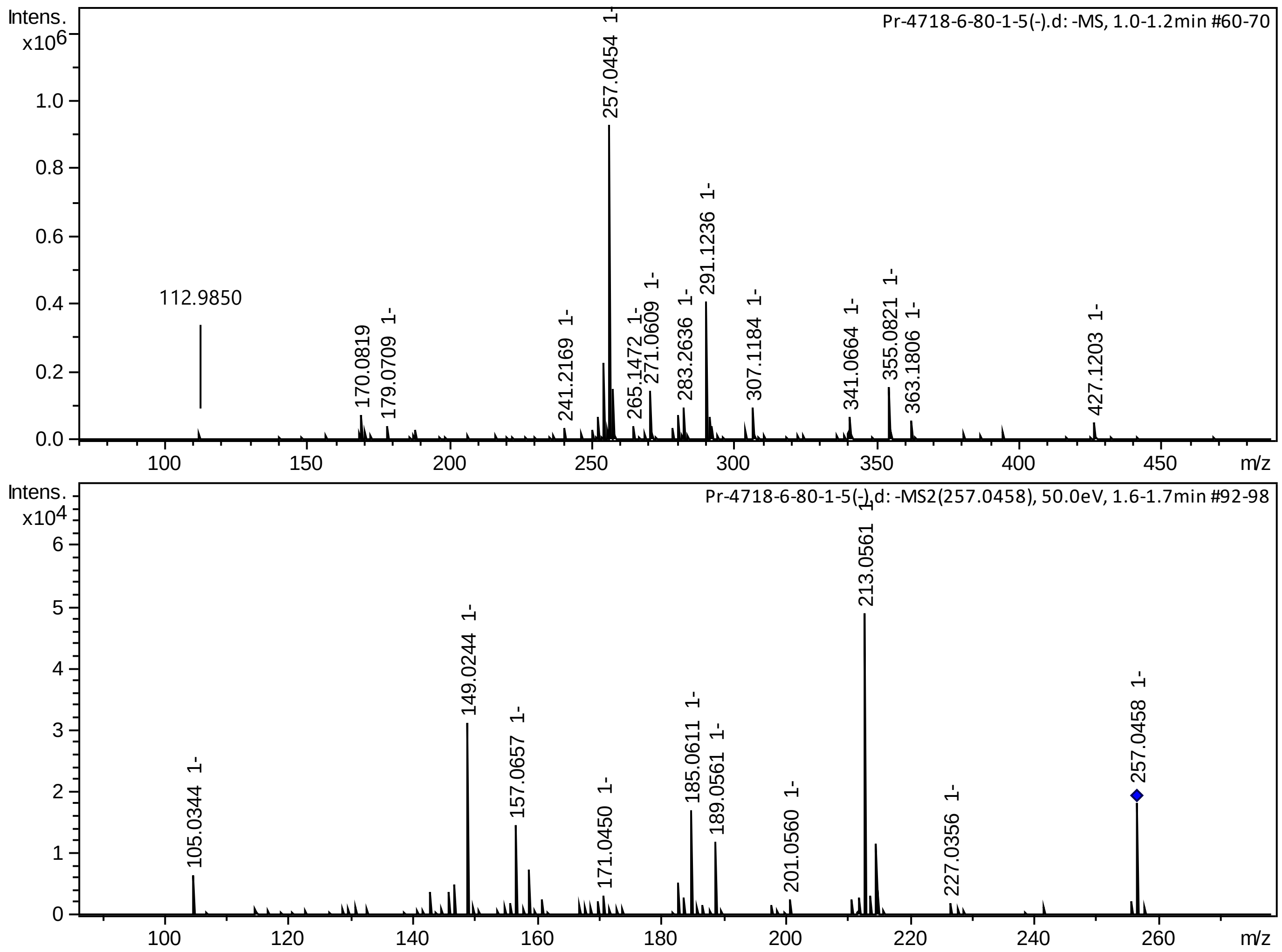
Intens.
x10^6
Pr-4718-6-80-1-5(-).d: -MS, 1.0-1.2min #60-70
1.0
0.8
0.6
0.4
0.2
0.0
112.9850
170.0819
179.0709 1-
241.2169 1-
257.0454 1-
265.1472 1-
271.0609 1-
283.2636 1-
291.1236 1-
307.1184 1-
341.0664 1-
355.0821 1-
363.1806 1-
427.1203 1-
100
150
200
250
300
350
400
450
m/z
Intens.
x10^4
Pr-4718-6-80-1-5(-).d: -MS2(257.0458), 50.0eV, 1.6-1.7min #92-98
6
5
4
3
2
1
0
105.0344 1-
149.0244 1-
157.0657 1-
171.0450 1-
185.0611 1-
189.0561 1-
201.0560 1-
213.0561 1-
227.0356 1-
257.0458 1-
100
120
140
160
180
200
220
240
260
m/z

Figure S40. ^1H **NMR spectrum of 11 measured at 500 MHz in acetone-**d_6

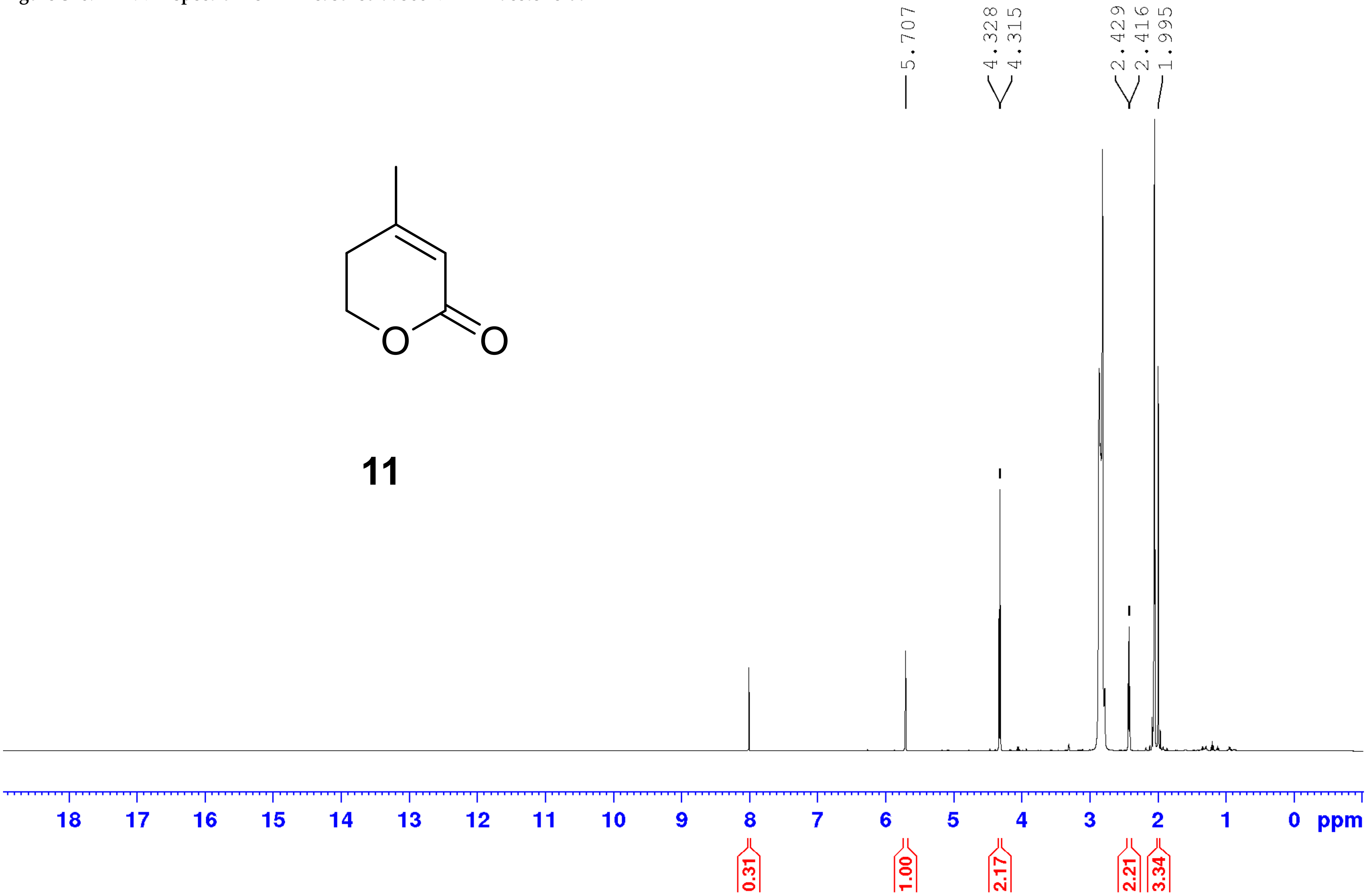

Figure S41. ^{13}**C NMR spectrum of 11 measured at 125 MHz in acetone-d**$_6$

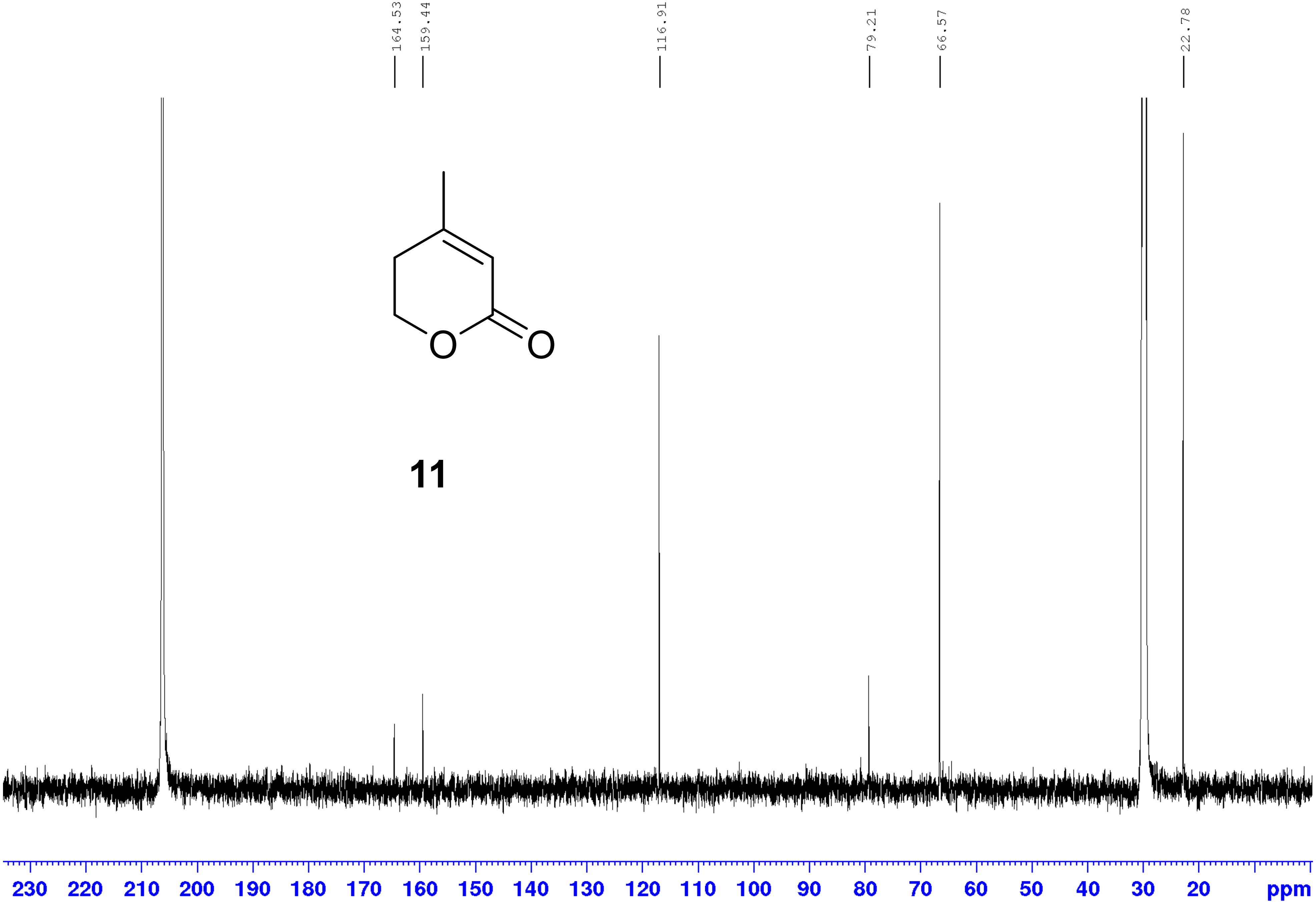

Figure S42. Two most stale conformations of *R*-1 calculated with B3LYP/cc-pvTz_PCM method.

Interaction between compounds under study with the solvent was accounted for at two levels of theory: first, using polarizable continuum model (PCM; “PCM level”), and second, via direct introduction to theoretical model of two methanol molecules (Direct; “Direct level”). At the “Direct level” the PCM approach was also used for modeling interaction of the clusters **1**&(CH_3OH)×2 and **2**&(CH_3OH)×2 with the solvent.

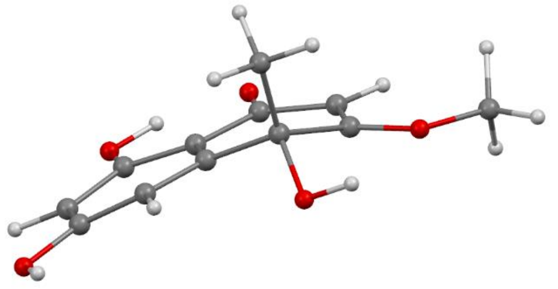

4R-**1_c1** **[g = 0.68]**

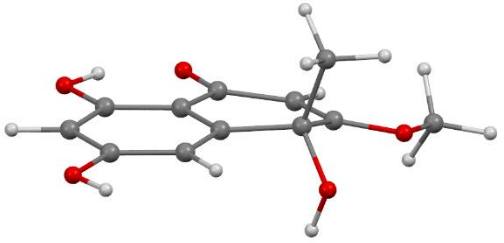

4R-**1_c2** **[g = 0.32]**

Figure S43. The optimized structures of 4*R*-1&(CH_3OH)×2.

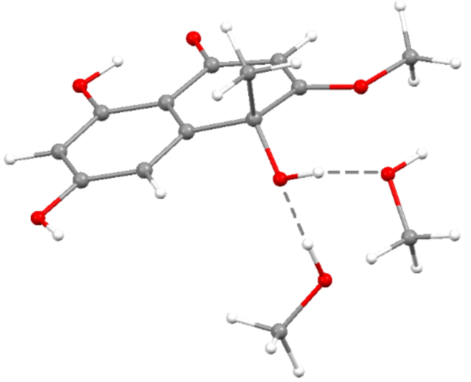

Direct_4*R*-**1_c1** [2.4%]

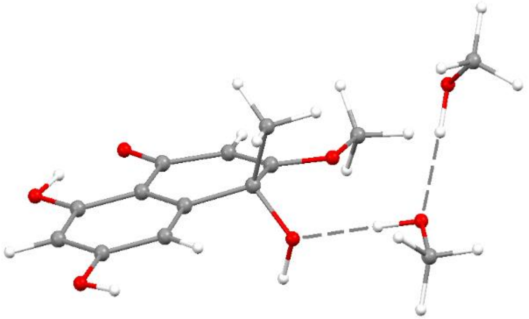

Direct_4*R*-**1_c2** [1.9%]

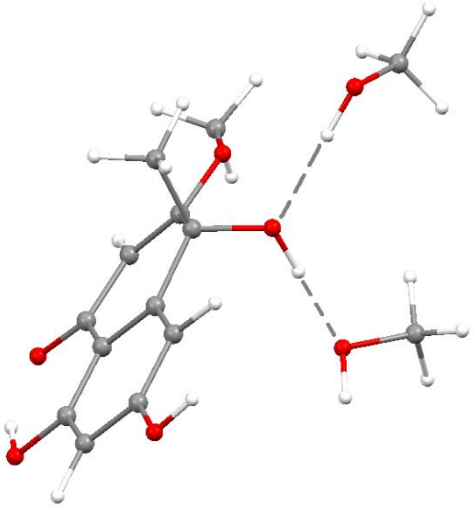 Direct_4*R*-**1_c3** [2.6%]	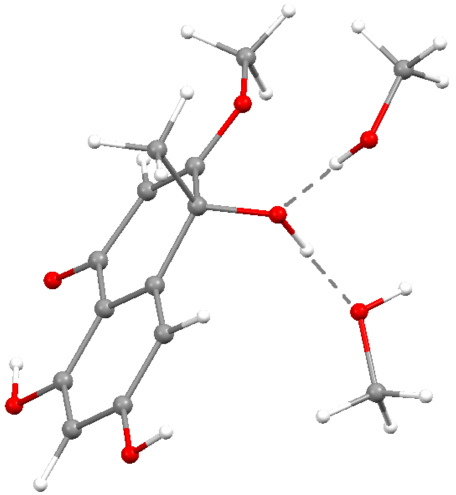 Direct_4*R*-**1_c4** [2.6%]
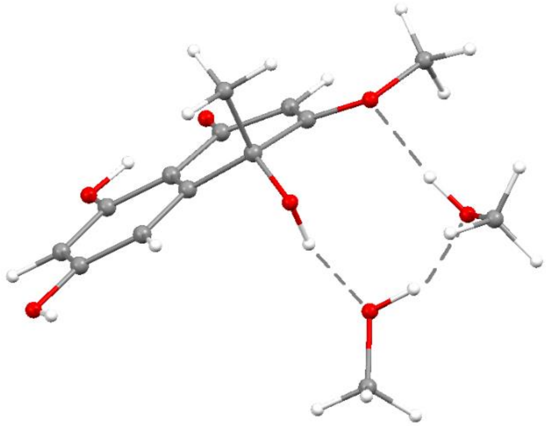 Direct_4*R*-**1_c5** [60.4%]	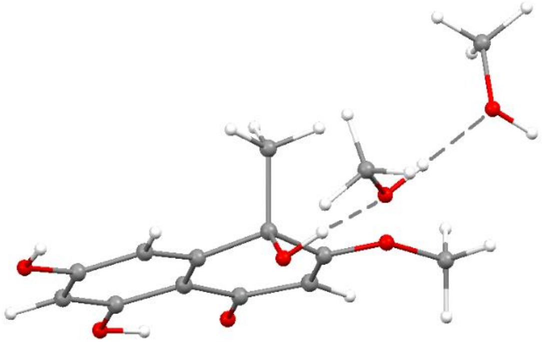 Direct_4*R*-**1_c6** [18.8%]

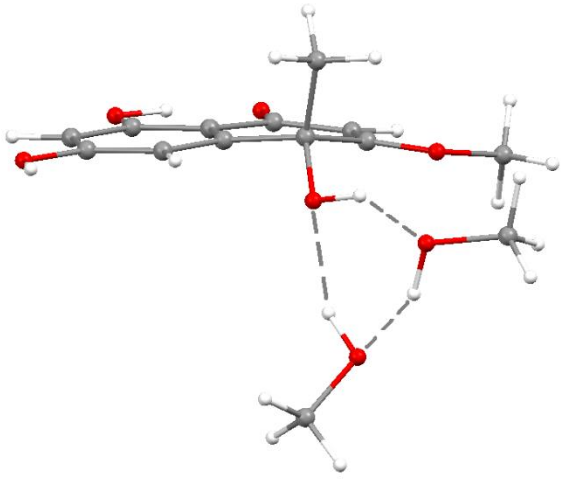

Direct_4*R*-**1_c7** [11.3%]	
